# Use of Mesh in Laparoscopic Paraesophageal Hernia Repair: A Meta-Analysis and Risk-Benefit Analysis

**DOI:** 10.1371/journal.pone.0139547

**Published:** 2015-10-15

**Authors:** Beat P. Müller-Stich, Hannes G. Kenngott, Matthias Gondan, Christian Stock, Georg R. Linke, Franziska Fritz, Felix Nickel, Markus K. Diener, Carsten N. Gutt, Moritz Wente, Markus W. Büchler, Lars Fischer

**Affiliations:** 1 Department of General, Visceral and Transplant Surgery, University of Heidelberg, Im Neuenheimer Feld 110, 69120, Heidelberg, Germany; 2 Department of Psychology, University of Copenhagen, Øster Farimagsgade 2A, 1315, København K, Denmark; 3 Institute of Medical Biometry and Informatics, University of Heidelberg, Im Neuenheimer Feld 305, 69120, Heidelberg, Germany; 4 The Study Center of the German Surgical Society, University of Heidelberg, Im Neuenheimer Feld 110, 69120, Heidelberg, Germany; 5 General, Visceral, Thoracic and Vascular Surgery, Klinikum Memmingen, Bismarckstraße 23, 87700, Memmingen, Germany; Heinrich-Heine-University and University Hospital Duesseldorf, GERMANY

## Abstract

**Introduction:**

Mesh augmentation seems to reduce recurrences following laparoscopic paraesophageal hernia repair (LPHR). However, there is an uncertain risk of mesh-associated complications. Risk-benefit analysis might solve the dilemma.

**Materials and Methods:**

A systematic literature search was performed to identify randomized controlled trials (RCTs) and observational clinical studies (OCSs) comparing laparoscopic mesh-augmented hiatoplasty (LMAH) with laparoscopic mesh-free hiatoplasty (LH) with regard to recurrences and complications. Random effects meta-analyses were performed to determine potential benefits of LMAH. All data regarding LMAH were used to estimate risk of mesh-associated complications. Risk-benefit analysis was performed using a Markov Monte Carlo decision-analytic model.

**Results:**

Meta-analysis of 3 RCTs and 9 OCSs including 915 patients revealed a significantly lower recurrence rate for LMAH compared to LH (pooled proportions, 12.1% vs. 20.5%; odds ratio (OR), 0.55; 95% confidence interval (CI), 0.34 to 0.89; p = 0.04). Complication rates were comparable in both groups (pooled proportions, 15.3% vs. 14.2%; OR, 1.02; 95% CI, 0.63 to 1.65; p = 0.94). The systematic review of LMAH data yielded a mesh-associated complication rate of 1.9% (41/2121; 95% CI, 1.3% to 2.5%) for those series reporting at least one mesh-associated complication. The Markov Monte Carlo decision-analytic model revealed a procedure-related mortality rate of 1.6% for LMAH and 1.8% for LH.

**Conclusions:**

Mesh application should be considered for LPHR because it reduces recurrences at least in the mid-term. Overall procedure-related complications and mortality seem to not be increased despite of potential mesh-associated complications.

## Introduction

Minimally invasive surgery has changed the surgical approach towards paraesophageal hernia repair. However, criticism has been raised because of recurrence rates of up to 42% [1]. As one possible approach to resolve the issue of recurrence, mesh augmentation has been proposed. Meanwhile several systematic reviews comparing use of mesh without the use of mesh have shown mesh augmentation to substantially reduce recurrence rates [2–5]. A drawback of mesh augmentation is a range of associated complications such as stenosis, migration and erosion [6]. A published survey of surgical practice concerning hiatal hernia (HH) repair reports the risk of mesh-associated complications to be about 0.5% [7]. Although occurring at a numerically low frequency, such mesh-associated complications are to be taken seriously as they might be fatal. Through own experience or from hearsay many surgeons have ambiguous and mostly negative associations when it comes to the concept of mesh augmentation at the hiatus. Therefore, they object to routine use of mesh for laparoscopic paraesophageal hernia repair (LPHR) [6,8,9]. On the other hand, it could be argued that mesh augmentation reduces fatal complications by preventing reoperation for recurrent HH.

A risk-benefit analysis, which addresses this controversy, might solve the dilemma. Such an analysis has not been conducted so far. A decision-tree analysis done by Obeid et al. showed the importance of recurrences and reoperation rate changing utility scores. However, no meta-analyses of recurrences and complications and no systematic review of mesh-associated complications were performed to obtain estimates for the relevant input variables of the decision-tree analysis [10].

The aims of the present study were therefore to systematically retrieve, appraise and analyze the existing literature on LPHR for relevant input variables such as rates of recurrences and complications and to explore in a Markov Monte Carlo decision-analytic simulation whether the benefits outweigh the risk of mesh augmentation in LPHR.

## Materials and Methods

The systematic review and meta-analysis were performed in accordance with the “Preferred Reporting Items for Systematic Reviews and Meta-Analysis” (PRISMA) statement [11]. No protocol has been published prior to this work.

### Search strategy and selection criteria

A systematic literature search was conducted independently by two authors (HGK and FF) using the method described by Haynes et al. [12]. The search strategy for Medline (Pubmed) was based on a combination of medical subject headings and keywords (Box 1). The database search included the Cochrane Central Register of Controlled Trials and Medline (1966 to July 2015). The search was restricted to English and German language studies. Cross-referencing and a manual search were used additionally. Included were systematic reviews, randomized controlled trials (RCTs) as well as prospective and retrospective observational clinical studies (OCSs) focusing on use of mesh for LPHR in adults. Animal studies, pediatric studies (age under 18 years), method outlines, comments, letters to the editor, duplicate publications and all non-systematic reviews were excluded. Any disagreements in the selection process were resolved by discussion with a third author (BPMS).

**Box 1. Search algorithm**

"Gastroesophageal Reflux"[Mesh] OR "Hernia, Hiatal"[Mesh] OR (("Hiatus"[tw] OR "Hiatal"[tw]) AND "Hernia"[tw]) AND ("Laparoscopy"[Mesh] OR "Laparoscop*"[tw] OR "minimally invasive"[tw] OR "Celioscop*"[tw] OR "Peritoneoscop*"[tw]) AND ("Surgical Mesh"[Mesh] OR "mesh"[tw])

### Data extraction

The results on all predefined outcomes of included trials were extracted independently by two authors (HGK and FF). Any disagreements in the selection process and data extraction were resolved by discussion with a third author (BPMS).

Quality of included studies was assessed independently by two authors (HGK and FF) and further discussed with a third author (BPMS) in the case of disagreement. For this critical appraisal a standardized form was created in accordance with international recommendations [13,14].

### Evidence synthesis

Three types of evidence synthesis were performed: (A) a meta-analysis of recurrence and complications, (B) a critically appraised synthesis of mesh-related complications including a proportion meta-analysis, and (C) a Markov Monte Carlo decision-analytic model simulation addressing the risks and benefits of laparoscopic mesh-augmented hiatoplasty (LMAH).

#### A. Meta-analysis of recurrence and complications

Meta-analysis was done to identify the type of surgical strategy that resulted in fewer anatomical recurrences and less overall operative complications in patients with paraesophageal hernia (PEH). PEH were defined as HH with paraesophageal involvement, which was postulated in large HH with a diameter of 5 cm and more. Out of the primary search as mentioned above, this meta-analysis focused exclusively on the RCTs and OCSs comparing LMAH to laparoscopic mesh-free hiatoplasty (LH), with a follow-up of at least 6 months and a minimum of 10 patients in each group. There was no limitation regarding the type of mesh used (shape, material). Recurrences had to be verified either by esophagogram or endoscopy. Articles not mentioning complications were excluded from the meta-analysis of overall operative complications since the risk of bias by underreporting was expected to be too high.

The comparison of the dichotomous primary outcome (recurrence yes/no) was carried out using a conventional Mantel-Haenszel random effects meta-analysis and is reported as an odds ratio (OR) with 95% confidence interval (95% CI) [15]. Heterogeneity of the study-specific results was quantified by the descriptive *I* ^2^ inconsistency measure [16]. Possible publication bias was inspected using funnel plots and asymmetry tests [17]. Estimates and 95% CIs for group-specific proportions of patients suffering from recurrences and complications were obtained by random-intercept meta-analysis of logit-transformed proportions, with DerSimonian-Laird estimator of the heterogeneity of the studies [18].

#### B. Systematic review of published mesh-associated complications

The primary objective of this section was to identify all published LMAH complications. Therefore, all published data retrieved by the above-mentioned primary search (without any restriction to e.g. a minimal follow-up period or a minimal number of patients included) was investigated for information on mesh-associated complications. The risk of mesh-associated complications was determined calculating three different rates. Firstly, all published LMAH and mesh-associated complications including case reports were pooled. Since case reports on mesh-related complications do not provide a reasonable estimate of the rate of complications in the mesh-treated population they were excluded for further calculations. Secondly, only series explicitly mentioning the occurrence of mesh-associated complications, and thirdly, series only mentioning at least one mesh-associated complication were considered for the calculation. Since the latter series led to the highest rate among the three calculated rates of mesh-associated complications, it was chosen as the estimate for the risk-benefit analysis in section C. This decision was made to compensate for potential publication bias and to thereby obtain the most conservative risk-benefit analysis. Estimates and 95% CIs were obtained by means of intercept-only logistic regression with robust standard errors.

#### C. Risk-benefit analysis

**Fig 1:**
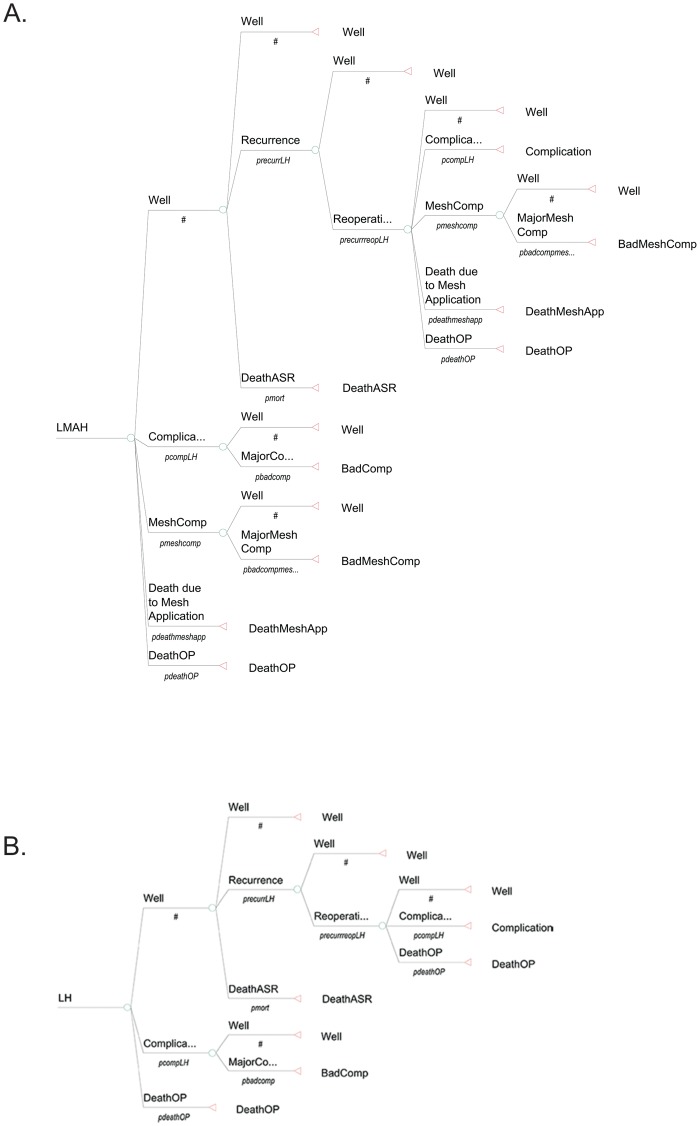
Decision tree analysis for the two treatment strategies A) LMAH vs. B) LH. (The cycles of BadComp and BadMeshComp are not shown in the figure. Patients entering these states stay inside them until they die of age- or gender-related causes.) LMAH, laparoscopic mesh-augmented hiatoplasty; LH, laparoscopic mesh-free hiatoplasty; Well, state of well-being; Complication, State of having a complication after surgery; DeathOP, Death due to the operation; DeathASR, Death due to age- and sex-related causes; MajorComp, state of having a major complication, leading to BadComp; BadComp, permanently lowered quality of life due to a major complication; MeshComp, state of having a mesh-associated complication; BadMeshComp, permanently lowered quality of life due to a major mesh-associated complication.

Structure of the model: Two different groups with 100.000 patients each were assessed and compared in a Markov cohort: LMAH and LH. In addition a Markov Monte Carlo decision-analytic model for 100.000 patients (risk-benefit analysis) [19–21] was developed following Stylopoulos et al. [22] using the TreeAge Pro Healthcare 2014 software (Treeage Software Inc., Williamstown, MA). Each cycle in the model represents one month, and the cohort is followed until death by any cause. Each operation has three possible immediate outcomes: recovery (Well), surgical morbidity (Complication) or surgical mortality (DeathOP). The basis of the decision-analytic model is the assumption that complications differently affect patients’ quality of life (QoL). As in Stylopoulos et al., the grading system proposed by Clavien et al. was used to differentiate between complications causing permanently reduced QoL (Clavien III) and minor complications (Clavien I+II) [22,23]. In cases of minor complications patients are assumed to fully recover. Major complications result in ongoing therapy and in a reduced QoL. Patients then stay in the complication state until they die of age- or sex-related causes. The ‘Well’ state in the LH group has three possible outcomes: Death from age or gender-related causes (DeathASR), staying well (Well) or anatomical recurrence (Recurrence). Reoperation may result in the state of being well (Well), complication (Complication), mesh complication (MeshComp) or lead to death due to operation (DeathOP). The state complication (Complication) may lead to either recovery and patients will subsequently be in a state of well-being (Well) again or may lead to major complications (MajorComp) resulting in a persistent lowered QoL (BadComp). In this case patients will stay in a lowered QoL state until they die of age or gender-related causes (DeathASR). In the LMAH group an operation or reoperation may additionally lead to the state of mesh-associated complication (MeshComp) or death due to mesh application (DeathMeshApp). The state mesh-associated complication (MeshComp) may then lead either to recovery and well-being (Well), to a persistently lowered QoL (BadSympMeshComp) in case of major complication or to death due to mesh application (DeathMeshApp). The endpoints of the study included the probabilities of several clinical events, which were associated with the two treatment arms. (Fig 1)

### Input variables for probabilities, quality of life and risk of death

(for better understanding some of the following results were pre-drawn from the results section and summarized in Table 1)

**Table 1:** Input variables for probabilities (p) in the Markov Monte Carlo decision-analytic simulation.

Variable	Variable name	Without mesh	With mesh
Complications	PcompLH / pcompLMAH	14.2%*	15.3%*
Mesh-associatedcomplications	pmeshcomp		2.0%^†^
Major complications in relation to mesh-associatedcomplications	pbadmeshcompmeshcomp		31.9%
			(29 Clavien III complications / 91 mesh-ass. complications)^‡^
Reoperationsfollowing HHrecurrences	precurrreoperationLH / precurrreoperationLMAH	39.0%	20.3%
		(30 reoperations / 77 HH recurrences)^§^	(12 reoperations / 59 HH recurrences)^§^
Death due to mesh application	pdeathduetomeshapplication		0.073%
			(4 fatal complications/ 5499 meshes)^†^

HH, hiatal hernia.

* According to A in the results section;

^†^ according to B in the results section;

^‡^ according to Table 2;

^§^ according to S1 Table.

**Fig 2:**
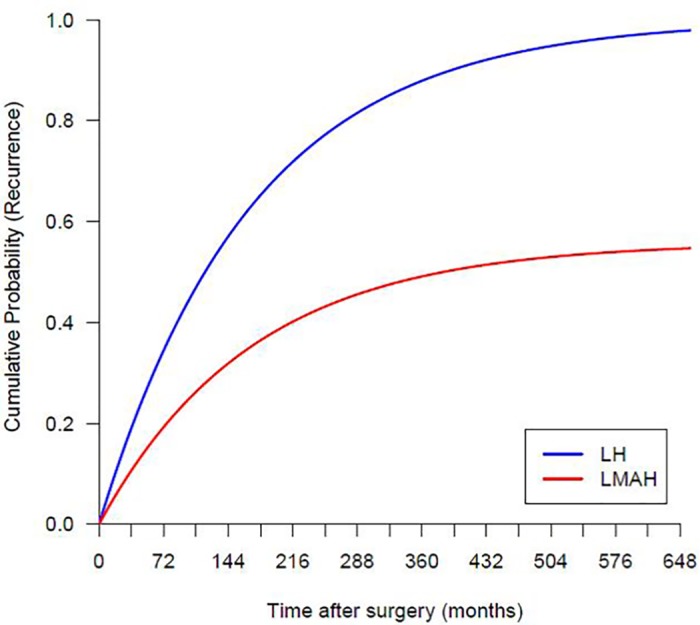
Cumulative probabilities of hiatal hernia recurrence calculated for the Markov Monte Carlo decision-analytic simulation.

The pooled proportions of recurrences were found to be 12.1% for LMAH and 20.5% for LH, thus LMAH equaling an incidence rate ratio of 59% (12.1%/20.5%) to LH, according to the results in section A. Monthly probabilities for recurrence of LH (*p*_recurrLH_) were derived from an exponential model for the cumulative recurrence of the form recurr_LH_ = 1−exp(−λ_LH_ * t), where λ_LH_ is a constant hazard and t denotes the time after surgery in months. The model was fitted through the origin and the pooled proportion of recurrences of LH at an overall mean length of follow-up of 34 months (S1 Table). It yielded a constant hazard of λ_LH_ = 0.006. The model implies higher recurrence rates in the first years after the operation but gradually decreasing rates for the subsequent years. Thereby it is likely to reflect reality. The cumulative recurrence for the LMAH group was calculated using the above stated incidence rate ratio as recurr_LMAH_ = 0.59 x recurr_LH_ and probabilities for recurrence of LMAH (*p*_recurrLMAH_) were derived from the cumulative recurrence. The monthly recurrence rates applied in the simulation model are shown in Fig 2. They are in line with long-term data from the literature [24,25].

Overall complication rates after LMAH and LH were estimated from the results of the meta-analysis in section A to be 15.3% (*p*_compLMAH_ = 0.153) and 14.2% (*p*_compLH_ = 0.142), respectively (S1 Table). The rate of mesh-associated complications was estimated to be 1.9% (*p*_meshcomp_ = 0.019) according to the results in Section B relating to studies with at least one mesh-associated complication (S1 and S2 Tables). The probability of having a major complication (Clavien III) in the postoperative course was estimated to be 1.94% (*p*_badcomp_ = 0.0194) irrespective of mesh use according to Stylopoulos et al. [22]. The probability of having a major complication (Clavien III) after a mesh-associated complication of 31.9% (29/91) (*p*_badcompmeshcomp_ = 0.319) was extracted from published mesh-associated complications and the documented surgical outcome (S1–S3 Tables). For reoperations the same complication rate was assumed as for primary surgery with the intention to not benefit LMAH. The probability of dying due to mesh application was calculated from the results in section A and B. There are four published deaths due to mesh application. Thus the risk of dying due to mesh application during an operation was estimated to 0.073% (p_deathmeshapp_ = 0.00073) (S1–S3 Tables), the probability of reoperation in relation to the occurrence of recurrent HH to 20.3% (12/59) (*p*_recurrreopLMAH_ = 0.203) for LMAH and 39.0% (30/77) (*p*_recurrreopLH_ = 0.390) for LH, extracted from the studies in section A (S1 Table). The age- and sex-adjusted mortality rate (*p*_mort_) was calculated from standard life tables provided by the Federal Statistical Office of Germany, increasing accordingly in each stage/month of the model [26]. In addition to age- and sex-adjusted overall mortality patients were exposed to operation-related mortality and excess mortality as a consequence of major complications (Clavien III). According to Stylopoulos et al. operation-related mortality was expected to be 1.38% (*p*_deathOP_ = 0.0138) [22].

For the computation of quality-adjusted life years (QALY) the method as it was used by Stylopoulos et al. for PEH analysis was also applied in this study [22]. The quality of well-being (QWB) index was used for QALY computations. QALYs were calculated in the form of the arithmetic product of life expectation and the respective QoL measure of the QWB. The QWB index ranges from 1.0 (asymptomatic optimum function) to 0.0 (death) [27]. QoL utility for patients without hernia (*u*_well_) were based on the mean of the QWB scores of persons without an impairing medical condition reported in the Beaver Dam Health Outcomes Study (*u*_well_ = 0.78 for a 65year-old patient) [28]. The utilities for patients with postoperative persistent diminished QoL (Clavien III) were derived from the same study (*u*_badLH_ = *u*_badLMAH_ = *u*_badmeshcomp_ = 0.64) [28]. The patients who were reoperated were believed to have the same QoL as those experiencing Clavien III complications due to hospitalization and decreased mobility for one cycle (*u*_reoperation_ = *u*_badLH_ = *u*_badLMAH_ = *u*_badmeshcomp_ = 0.64). Due to that estimation, no further disutilities for reoperations were used, since one month was expected to be enough for full recovery. In the case of complications, patients transfer accordingly to the state with lower utilities.

## Results

### Search results

**Fig 3:**
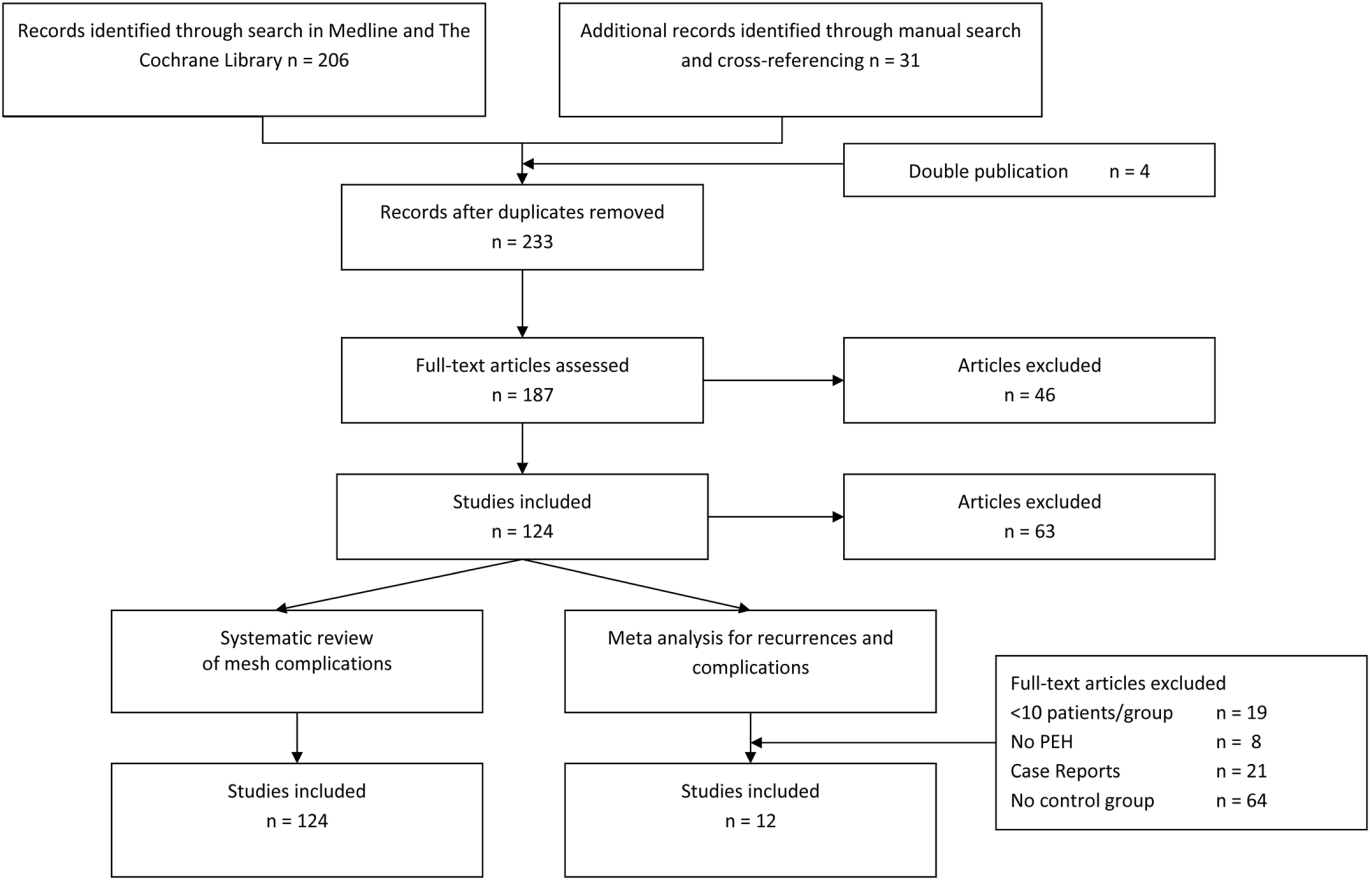
Flow chart of study selection.

The systematic review based on our search strategy yielded 119 studies with a total of 5499 patients. Fig 3 details the search and selection process including the reasons for the exclusion of articles. For the meta-analysis of recurrences 12 studies (3 RCTs and 9 OCSs) with 915 patients met the inclusion criteria. The meta-analysis of complications was based on 3 RCTs and 6 OCSs with 638 patients in total (S1 Table).

### Study quality and critical appraisal

The randomization procedure and allocation concealment of group allocation was adequately described in the RCT of Oelschlager et al. and Watson et al. [29–31]. Frantzides et al. however did not clearly describe the randomization strategy [32]. All RCT were neither patient nor assessor blinded. Clinical heterogeneity between the trials and the studies was substantial. The time to follow-up was not stratified between LMAH and LH in all OCS. Studies used different mesh material, mesh shape and fundoplication was not performed in all patients alongside differing in techniques and circumference. Definition of HH was neither homogenous between the studies. However, study populations were described adequately. The applied surgical technique was reported in a standardized manner, and a standardized follow-up of at least 6 months was performed in all studies. Follow-up varied highly between the studies. Objectives and outcomes were adequately defined in all trials and studies.

### A. Meta-analysis of recurrences and complications

#### Meta-analysis of recurrences

**Fig 4:**
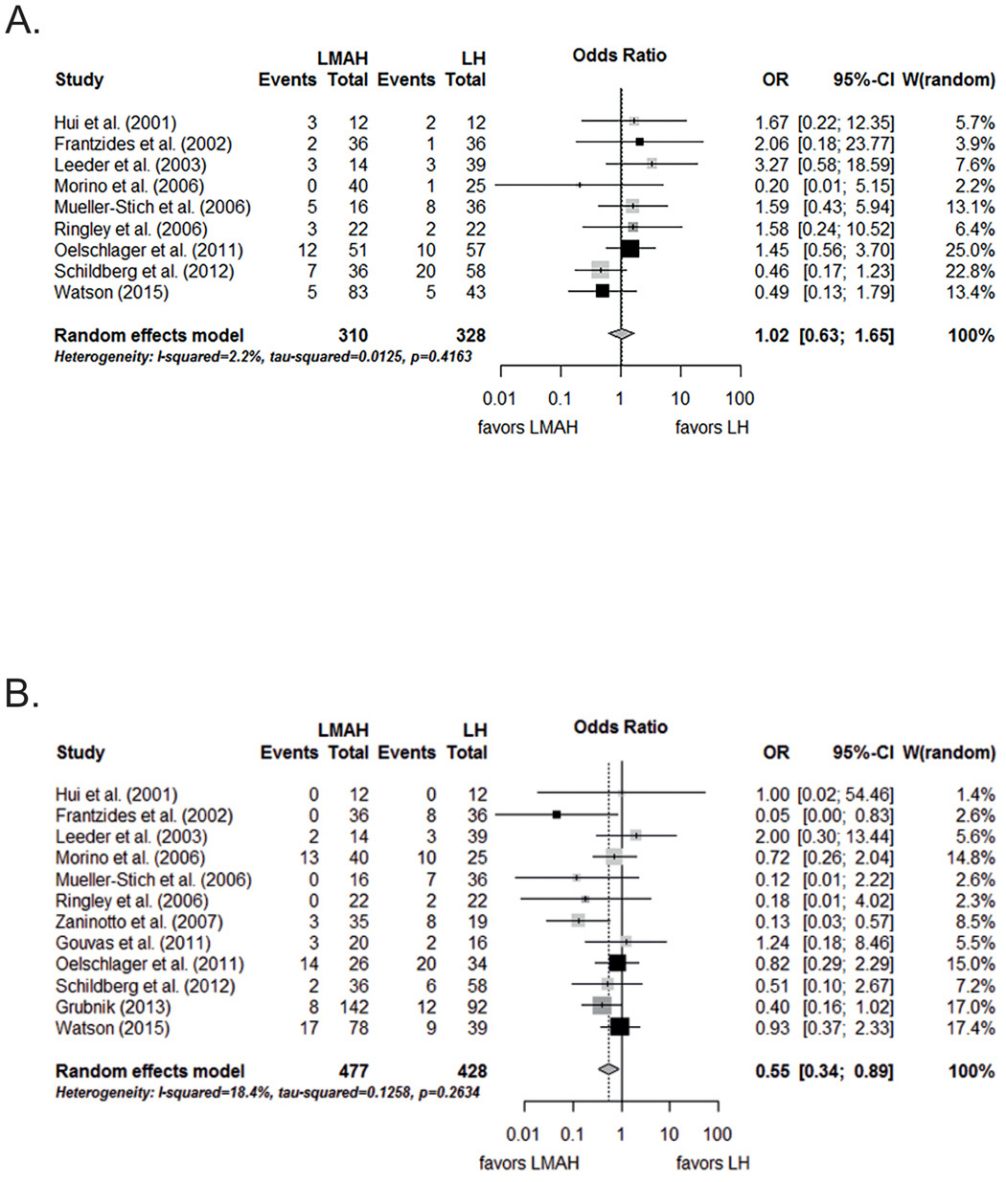
Meta-analysis of A) hernia recurrence and B) complications after LMAH and LH. Black rectangles are randomized controlled trials; dark gray rectangles are case control studies; light gray rectangles are case series with control group. LMAH, laparoscopic mesh augmented hiatoplasty; LH, laparoscopic hiatoplasty; OR, Odds Ratio; 95% CI, 95% confidence interval. Studies included in the meta-analysis are detailed in the supporting information files (S1 Table).

Fig 4A shows the significant reduction of recurrences after LMAH compared to LH after an overall mean length of follow-up of 34 months (S1 Table) (OR, 0.55; 95% CI, 0.34 to 0.89, p = 0.04). Qualitative and formal inspection of the funnel plot did not reveal publication bias (*t* = -0.81, *p* = 0.44). The pooled proportions of recurrence were 12.1% (95% CI, 6.3% to 22.2%) for LMAH and 20.5% (95% CI, 12.9% to 31.0%) for LH.

**Fig 5:**
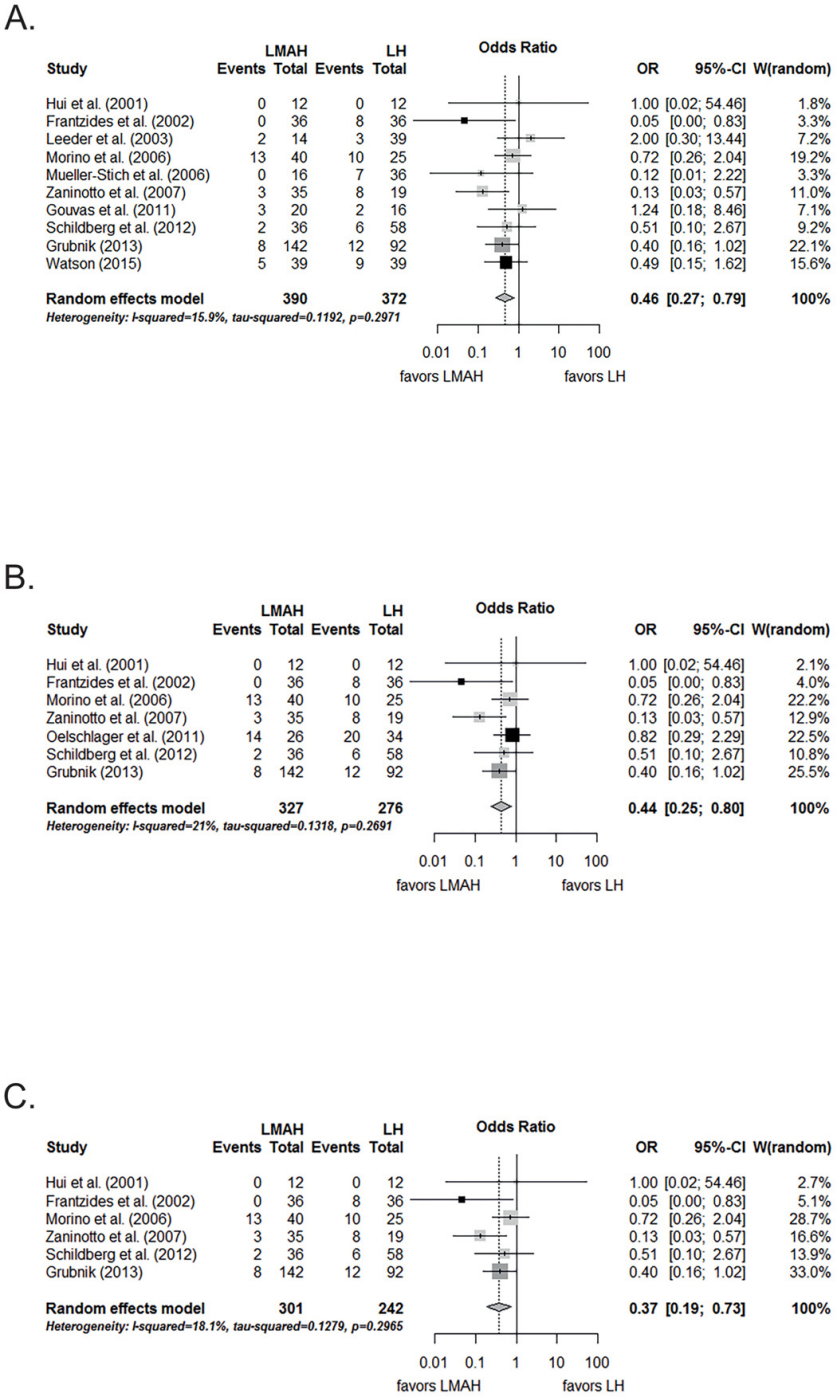
Meta-analysis of A) hernia recurrence for studies using synthetic meshes, B) for studies with at least 2 years of follow-up, C) for studies with both synthetic meshes and at least 2 years of follwow-up after LMAH and LH. Black rectangles are randomized controlled trials; dark gray rectangles are case control studies; light gray rectangles are case series with control group. LMAH, laparoscopic mesh augmented hiatoplasty; LH, laparoscopic hiatoplasty; OR, Odds Ratio; 95% CI, 95% confidence interval. Studies included in the meta-analysis are detailed in the supporting information files (S1 Table).

When only the studies that used synthetic meshes were assessed, the difference was still significant and in favor of LMAH when compared to LH (OR, 0.46; 95% CI, 0.27 to 0.79, p = 0.005; Fig 5A). The pooled proportions of recurrences were 9.9% (95% CI, 5.3% to 17.7%) for LMAH and 19.0% (95% CI, 12.8% to 27.2%) for LH.

When the meta-analysis included only studies with a follow-up period longer than 2 years for both study arms, the reduction of recurrences in the LMAH group was significant as well, when compared to the LH group (OR, 0.44; 95% CI, 0.25 to 0.80, p = 0.007; Fig 5B). The pooled proportions of recurrences were 11.5% (95% CI, 3.9% to 29.3%) for LMAH and 25.4% (95% CI, 13.1% to 43.4%) for LH.

Restricting the meta-analysis to studies investigating synthetic meshes with a follow-up period longer than 2 years in both study arms, again, a lower recurrence rate was found in the LMAH group compared to the LH group (OR, 0.37; 95% CI, 0.19 to 0.73, p = 0.004; Fig 5C). The pooled proportions of recurrence were 8.0% (95% CI, 2.9% to 20.1%) for LMAH and 20.9% (95% CI, 11.5% to 35.0%) for LH.

#### Meta-analysis of complications

As shown in Fig 4B, there was no significant difference between LMAH and LH (OR, 1.02; 95% CI 0.63 to 1.65; p = 0.94) in terms of complications. The test for publication bias did again not reveal evidence for asymmetry (*t* = 0.37, *p* = 0.72). Pooled proportions of complications were 15.3% (95% CI, 9.4% to 23.9%) for LMAH and 14.2% (95% CI, 8.5% to 22.7%) for LH.

When only the studies that used synthetic meshes were assessed complications rates still were similar in both groups (OR, 1.00; 95% CI, 0.56 to 1.81, p = 0.99). The pooled proportions of complications were 16.0% (95% CI, 9.4% to 25.9%) for LMAH and 13.5% (95% CI, 6.9% to 25.0%) for LH.

Similar results were found when the meta-analysis was restricted to studies with a follow-up period longer than 2 years in both study arms (OR, 0.90; 95% CI, 0.46 to 1.76, p = 0.76). The pooled proportions of complications were 15.3% (95% CI, 7.8% to 27.9%) for LMAH and 14.6% (95% CI, 6.3% to 30.2%) for LH.

Restricting the meta-analysis to studies investigating synthetic meshes with a follow-up period longer than 2 years in both study arms complication rates were equal in both groups (OR, 0.63; 95% CI, 0.28 to 1.42, p = 0.27). The pooled proportions of complications were 11.6% (95% CI, 4.3% to 28.0%) for LMAH and 11.6% (95% CI, 2.9% to 36.4%) for LH.

### B. Systematic review of mesh-associated complications

**Fig 6:**
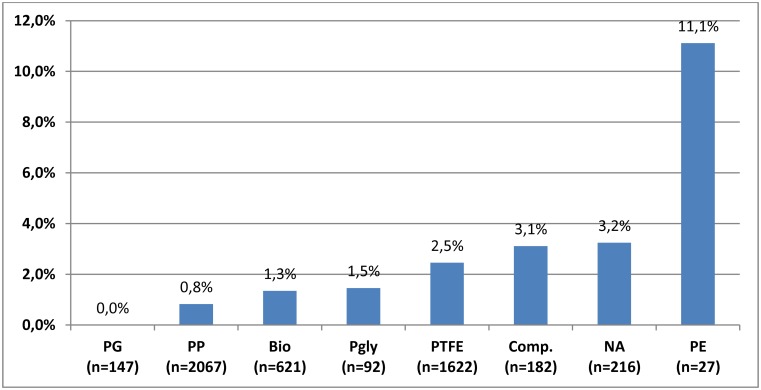
Mesh material-related risk of mesh associated complications. Comp., Composite; DM, dermal matrix; PE, polyethylene; PG, polyglactine; PGly; polyglycane; PP, polypropylene; PTFE, polytetrafluorethylene; SIS, small intestinal submucosa; NA, not answered.

A total of 5499 patients with LMAH were reported in 124 studies including 19 case reports (S1–S3 Tables). In total, 91 mesh-associated complications were reported; 50 in case reports and 41 in RCTs and OCSs. The main reasons for mesh-associated complications were erosions of the esophagus, the stomach or the aorta followed by stenoses and cardiac tamponades (Table 2). There were four fatal complications observed, all originating from cardiac tamponades caused by staples used for the fixation of the mesh to the hiatus (Table 2) [33–35]. When all mesh-associated complications found were related to all LMAH procedures published so far, including case reports, the mean rate of mesh-associated complications was 1.7% (91/5499; 95% CI, 1.4% to 2.0%). Considering only those series explicitly mentioning mesh-associated complications the complication rate was 0.9% (41/4477; 95% CI, 0.6% to 1.2%) and 1.9% (41/2121; 95% CI, 1.3% to 2.5%) for those series reporting at least one mesh-associated complication. 71.5% (3934/5499) of the patients received either polypropylene (PP) or polytetrafluorethylene (PTFE) meshes. PP meshes were used in 39.6% (2181/5499) and 31.9% (1753/5499) of all patients received PTFE meshes with a respective complication rate of 0.8% (18/2181, 95% CI 0.4% to 1.2%) and 2.5% (43/1753; 95% CI 1.8% to 3.2%). Biological meshes were implanted in 13.5% (744/5499) of reported patients with a complications rate of 1.3% (10/744; 95% CI, 0.5% to 2.1%) (Fig 6). The risk of dying of an operation due to mesh application was 0.073% (4/5499).

**Table 2:** Summary of the different types of mesh-associated complications (according to S1–S3 Tables).

Mesh complications	N	Clavien grade I + II	Clavien grade III	Missing data
Stenosis	27	19	7	1
Erosions	49	19	16	13
Fistula	1	1	0	0
Cardiac tamponade	8	2	5	1
Fibrosis	5	5	0	0
Aortic lesion	2	1	1	0
Total	91	47	29	15

### C. Risk-benefit analysis of LMAH

The Markov Monte Carlo decision-analytic model simulation showed similar QALYs for LMAH and LH (19.27 vs. 19.23). In the LMAH group 1.61% (1.606/100.000) died of surgery. Among these 0.12% (119/100.000) died of a reoperation and 0.079% (79/100.000) died due to mesh application. In the LH group 1.79% (1.790/100.000) died of surgery. Among these 0.41% (410/100.000) died of a reoperation.

## Discussion

To our knowledge, this is the first systematic review with meta-analysis showing that the benefits of mesh use in LPHR outweigh its risks. Based on previously published data the present meta-analysis reveals nearly a bisection of recurrences from 20.5% to 12.1% after a follow-up period of approximately three years. Mesh-associated complications are rare at a rate of 1.9% and do not markedly contribute to overall procedure-related complications.

The pooled proportions of recurrences found in the present meta-analysis are not as high as supposed by Hashemi et al. who reported 42% (1). However, the reduction from 20.5% to 12.1% after use of mesh corresponds to an absolute risk reduction of 8.4% and a number needed to treat of 12 (95% CI, 10.6 to 13.5). Of note, there was no publication bias in favor of the mesh group according to the funnel plot analysis. It is evident that such a risk reduction may lead to fewer reoperations. To estimate the number of reoperations that could be prevented during the first three years after primary surgery with use of mesh, the pooled proportions of recurrences obtained from the present meta-analysis can be multiplied by the rate of expected reoperations per recurrence. According to literature, this affects 20% of mesh patients and 39% of non-mesh patients (Table 1). Based on this, estimated reoperation rates after LMAH and LH are 2.4% and 8.0%, respectively, and correspond with an absolute risk reduction of 5.6% and a number needed to treat of 18 (95% CI, 13.3 to 27.3). Considering that risky reoperations are more than three-fold increased after LH, it is not surprising that the risk-benefit analysis revealed an 11% higher life-long operation-related mortality rate for LH (1.61% vs. 1.79%) corresponding to an absolute risk reduction of 0.3% and a number needed to treat of 344 (95% CI, 297.6 to 406.5). Furthermore, it has to be taken into account that, due to missing data for LPHR, in the present analysis the risk of reoperation was estimated to be the same as that of primary surgery in order not to benefit LMAH. However, there is evidence that even after antireflux surgery mortality is tenfold increased for redo-surgery than for primary surgery [36].

Due to several limitations the presented data has to be considered with caution. It might be argued that the difference of recurrences between the treatment groups was related to a follow-up period that was too short. However, a follow-up of approximately three years in both treatment groups can be considered reasonable for the detection of a relevant percentage of recurrences. Accordingly, our subgroup analysis restricted to studies with follow-up periods longer than 2 years showed a similar reduction of recurrences in the LMAH group compared to the LH group from 25.4% to 11.5% (OR, 0.44; 95% CI, 0.25 to 0.80, p = 0.007). In line with that, Hashemi et al. found a recurrence rate of 42% after a median follow-up of only 17 months [1]. Additionally, Zaninotto et al. who performed a consecutive endoscopy every two years over a median follow-up period of 64 months reported that all recurrences in their mesh patients occurred within the first eight postoperative months [25]. Other authors who reported findings after longer follow-up periods performed just one single radiographic examination in every patient at different time points after surgery. This way, however, the exact points in time of recurrences cannot be reconstructed and a late recurrence could have developed much earlier [24,37].

Furthermore, one might criticize that a publication bias cannot be excluded as a reason for the low rate of mesh-associated complications of approximately 2% and the calculated related mortality of 0.079% according to the present analysis. In fact, it is true that based on existing evidence, it is not possible to define the exact rate of mesh-associated complications. For this we would need reliable prospective register-based studies, which do not exist so far. On the other hand the risk of mesh-associated complications cannot be estimated based on reports of complication series as published by Stadlhuber et al. [6] because in this setting the underlying number of meshes implanted is missing. In our opinion, a more suitable method to estimate the risk of mesh-associated complications, is to correlate the number of published complications to the number of published implanted meshes in a reasonable way. With the intention to compensate for a potential publication bias in favor of LMAH (no publishing of mesh-associated complications) we chose the highest risk of mesh-associated complications found by this method for our risk-benefit analysis considering only studies with at least one mesh-associated complication. The 1.9% rate of mesh-associated complications found by this approach indeed seems to be conservative, since it has been compared doubled to the findings of the two biggest consecutive patient series ever published. Both of them found a rate of about 1% [9,38]. Given that the survey of the Society of American Gastrointestinal and Endoscopic Surgeons did not find mesh-associated complication rates higher than 0.5%, our estimate can be considered a conservative upper bound of the true complication rate. One might object that longer follow-up periods would detect much higher mesh-associated complication rates due to migration and erosion. However, approximately 90% of mesh complications occur within two years after surgery [6,39] and the mean overall follow-up rate of the controlled studies included in the present meta-analysis was close to three years.

Additionally, it has been argued that a relevant percentage of mesh-associated complications require operative revision if not even major organ resections, which is not justifiable in the setting of a benign disease. Parker et al. compared 68 patients with revisional foregut surgery and found a need for major resection in 30% of patients following mesh use compared to 4% in the non-mesh group. In absolute numbers this meant 3 major resections in 10 mesh patients compared to 3 major resection in 68 non-mesh patients [8]. Unfortunately, it is not clear whether the two groups were truly comparable. No information was provided regarding the mesh experience of the surgeons who implanted the 10 meshes or those who performed the revisional surgery. Additionally, selection bias could have been a potential source of bias. For example more complex cases could have been referred to the authors’ center and may have resulted in major complications. In fact, to date we have too little information on potential courses after revisional foregut surgery, neither for mesh patients nor for non-mesh patients, to make a reliable conclusion in this regard.

Finally, it is a limitation of the present analysis that different mesh materials, mesh shapes, operative techniques, sizes of HH, comorbidities, surgeon’s expertise and patient age were included without stratification for subgroups. Furthermore, the data quality of included studies varied extensively. Despite this, the main finding of the present study remains the same: Meshes reduce the recurrence rate following primary laparoscopic repair of PEH at least in the mid-term. Different types of meshes may have this effect to a larger or lesser extent. Whether a certain mesh type is superior in the prevention of recurrences cannot be concluded from the present data. However, it is remarkable that the only controlled trial investigating biological meshes did not show them to be beneficial with regard to the prevention of recurrences [30]. Anyhow, restricting the meta-analysis to studies investigating only synthetic meshes did not reveal a distinctly more prominent effect in terms of recurrence reduction (OR, 0.46; 95% CI, 0.27 to 0.79, p = 0.005). Only when the subgroup analysis was focused on synthetic meshes combined with a follow-op period longer than 2 years did a more prominent effect become apparent in favor of synthetic meshes with a reduction of recurrences from 20.9% to 8.0% (OR, 0.37; 95% CI, 0.19 to 0.73, p = 0.004). This illustrates that more well-designed and randomized controlled studies are needed to uncover the specific benefits of different mesh types in terms of their potential to reduce the number of recurrences.

Considering mesh materials, especially PP and PTFE meshes were assumed to induce more complications, so far [2,7]. However, it should be considered that, according to our analysis, PP and PTFE accounted for three forth of implanted meshes. Thus, it is evident that a higher absolute number of implanted meshes induces a higher absolute number of complications. Expressed in terms of percentages, the present analysis revealed the second-lowest mesh-associated complication rate of 0.8% for PP. This finding might be due to a quick, solid and stable tissue integration of PP meshes [38–40]. In contrast, biological meshes had an even higher complication rate of 1.3% in the present analysis (Fig 6). However, this might be due to the novelty of the products and the associated learning curve. At any rate, it seems that multiple factors such as mesh materials, mesh structure, material compositions, shape and kind of application contribute to the effectiveness and the risk of meshes [40–44]. Further studies are needed to find the safest and most effective mesh repair at the hiatus.

In summary, based on the above presented data it can be concluded that mesh use for LPHR should at least be considered. It seems to have a positive effect on the prevention of recurrences at least in the mid-term and, as a consequence, may reduce required reoperations without increasing the risk of overall procedure-related complications and mortality. Further well-designed studies will help to improve patient selection, operative technique and choice of mesh type as to even enhance the risk-benefit profile.

## Supporting Information

S1 PRISMA Checklist PRISMA Checklist. (DOC)

S1 Table Summary of trials and studies included in the meta-analysis (DOCX)

S2 Table Studies included in the systematic review of mesh-associated complications (observational clinical studies) (DOCX)

S3 Table Studies included in the systematic review of mesh-associated complications (case reports) (DOCX)
