# Correction: Use of Mesh in Laparoscopic Paraesophageal Hernia Repair: A Meta-Analysis and Risk-Benefit Analysis

**DOI:** 10.1371/journal.pone.0171865

**Published:** 2017-02-03

**Authors:** Beat P. Müller-Stich, Hannes G. Kenngott, Matthias Gondan, Christian Stock, Georg R. Linke, Franziska Fritz, Felix Nickel, Markus K. Diener, Carsten N. Gutt, Moritz Wente, Markus W. Büchler, Lars Fischer

In Fig 4, the images for panel A and panel B are switched. Please see the correct Fig 4 and its caption here.

**Fig 4:**
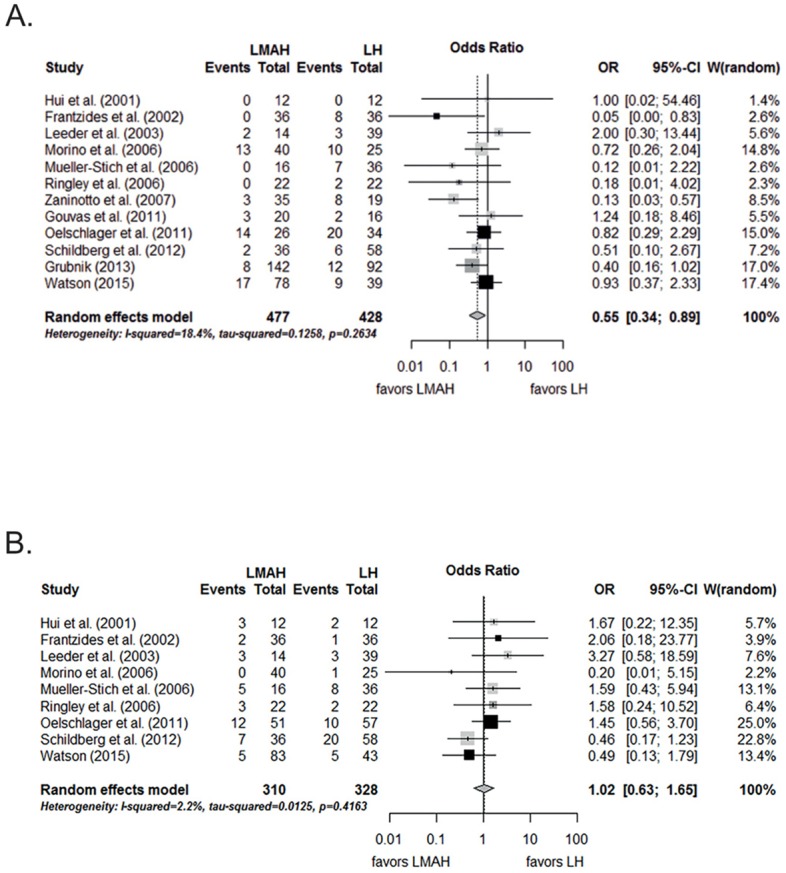
Meta-analysis of A) hernia recurrence and B) complications after LMAH and LH. Black rectangles are randomized controlled trials; dark gray rectangles are case control studies; light gray rectangles are case series with control group. LMAH, laparoscopic mesh augmented hiatoplasty; LH, laparoscopic hiatoplasty; OR, Odds Ratio; 95% CI, 95% confidence interval. Studies included in the meta-analysis are detailed in the supporting information files (S1 Table).
