# Supplementary material for: Use of Mesh in Laparoscopic Paraesophageal Hernia Repair: A Meta-Analysis and Risk-Benefit Analysis
Source: PLoS One. 2015 Oct 15;10(10):e0139547. doi: 10.1371/journal.pone.0139547 (PMC4607492; doi:10.1371/journal.pone.0139547)
Supplement: S1 Table — (DOCX) [file pone.0139547.s002.docx]

| **Supporting Information**  **S1 Table.** Summary of trials and studies included in the meta-analysis | | | | | | | | | | |
| --- | --- | --- | --- | --- | --- | --- | --- | --- | --- | --- |
| **Study** | **Year** | **N** | **Design .**  **Population** | **Fundo-plication**  **[degrees]** | **Mesh shape** | **Mesh material** | **Follow-up**  **[months]** | **Outcome parameters** | | |
|  |  |  |  |  |  |  |  |  | **Without mesh**  **[n/N (%)]** | **With mesh**  **[n/N (%)]** |

| **Randomized controlled trials** | | | | | | | | | | |
| --- | --- | --- | --- | --- | --- | --- | --- | --- | --- | --- |
| Frantzides et al. [[1](#_ENREF_1)] | 2002 | 72 | RCT-SC  LHH | 360 | Circ. | PTFE | 40 ± 20 | Symptomatic recurrences | 8/36 (22) | 0/36 (0) |
|  |  |  |  |  |  |  |  | Anatomical recurrences | 8/36 (22) | 0/36 (0) |
|  |  |  |  |  |  |  |  | Complications  Mesh-associated | 1/36 (3) | 2/36 (6)  0/36 (0) |
|  |  |  |  |  |  |  |  | Reoperations | 5/36 (14) | 0/36 (0) |
| Oelschlager et al. [[2](#_ENREF_2), [3](#_ENREF_3)] | 2006*  2011^†^ | 108 | RCT-MC  PEH | 360 | U | SIS | 59 ± 10^†^ (no mesh)  60 ± 10^†^ (mesh) | Symptomatic recurrences | NA | NA |
|  |  |  |  |  |  |  |  | Anatomical recurrences | 20/34 (59)^†^ | 14/26 (54)^†^ |
|  |  |  |  |  |  |  |  | Complications  Mesh-associated | 10/57 (18)* | 12/51 (24)*  0/51 (0)* |
|  |  |  |  |  |  |  |  | Reoperations | 2/57 (4)^†^ | 0/33 (0)^†^ |
| Watson et al. [[4](#_ENREF_4)] | 2015 | 126 | RCT-MC  LHH | Mixed | Rect. | SIS (41)  PP (42) | 6 | Symptomatic recurrences | NA | NA |
|  |  |  |  |  |  |  | 6 | Anatomical recurrences | 9/39 (23) | 17/78 (22) |
|  |  |  |  |  |  |  | 6 | Complications  Mesh-associated | 9/43 (21) | 8/83 (10) |
|  |  |  |  |  |  |  | 12 | Reoperations | 4/43 (7) | 1/78 (1) |

| **Observational clinical studies** | | | | | | | | | | |
| --- | --- | --- | --- | --- | --- | --- | --- | --- | --- | --- |
| Ringley et al. [[5](#_ENREF_5)] | 2006 | 44 | CT-CCS  LHH | 360 | U | DM | 12 (8-28)  (no mesh)  7 (3-12)  (mesh) | Symptomatic recurrences | NA | NA |
|  |  |  |  |  |  |  |  | Anatomical recurrences | 2/22 (9) | 0/22 (0) |
|  |  |  |  |  |  |  |  | Complications  Mesh-associated | 2/22 (9) | 3/22 (14)  0/22 (0) |
|  |  |  |  |  |  |  |  | Reoperations | 0/22 (0) | 0/22 (0) |
| Hui et al. [[6](#_ENREF_6)] | 2001 | 24 | SEC-SC  PEH | 360 | Rect. | PTFE(8)PP (4) | 37 (24-48) | Symptomatic recurrences | 0/12 (0) | 0/12 (0) |
|  |  |  |  |  |  |  |  | Anatomical recurrences | 0/12 (0) | 0/12 (0) |
|  |  |  |  |  |  |  |  | Complications  Mesh-associated | 2/12 (17) | 3/12 (25)  0/12 (0) |
|  |  |  |  |  |  |  |  | Reoperations | 0/12 (0) | 1/12 (8) |
| Leeder et al. [[7](#_ENREF_7)] | 2003 | 53 | SEC-SC  PEH | 360  180 | U | PP | 15 (6-52)  (no mesh)  46 (18-89)  (mesh) | Symptomatic recurrences | 3/39 (8) | 2/14 (14) |
|  |  |  |  |  |  |  |  | Anatomical recurrences | NA | NA |
|  |  |  |  |  |  |  |  | Complications  Mesh-associated | 3/39 (8) | 3/14 (21)  0/14 (0) |
|  |  |  |  |  |  |  |  | Reoperations | 2/39 (14) | 1/14 (7) |

| Müller-Stich et al. [[8](#_ENREF_8)] | 2006 | 56 | SEC-SC  PEH | 360  270  180 | Butterfly-shaped | PP | 67 (9-117)  (no mesh)  20 (10-60)  (mesh) | Symptomatic recurrences | 4/36 (11) | 0/16 (0) |
| --- | --- | --- | --- | --- | --- | --- | --- | --- | --- | --- |
|  |  |  |  |  |  |  |  | Anatomical recurrences | 7/36 (19) | 0/16 (0) |
|  |  |  |  |  |  |  |  | Complications  Mesh-associated | 8/36 (22) | 5/16 (31)  0/16 (0) |
|  |  |  |  |  |  |  |  | Reoperations | 2/36 (6) | 0/16 (0) |
| Morino et al. [[9](#_ENREF_9)] | 2006 | 65 | SEC-SC  LHH  PEH | 360 | U | PTFE(4)  PP (26)  Comp. (10) | 36 | Symptomatic recurrences | NA | NA |
|  |  |  |  |  |  |  |  | Anatomical recurrences | 10/25 (40) | 13/40 (33) |
|  |  |  |  |  |  |  |  | Complications  Mesh-associated | 1/25 (4) | 0/40 (0)  0/40 (0) |
|  |  |  |  |  |  |  |  | Reoperations | 6/25 (24) | 5/40 (13) |
| Zaninotto et al. [[10](#_ENREF_10)] | 2007 | 54 | SEC-SC  PEH | 360  270 | Circ. | Comp. | 64 (6-104)  (no mesh)  33 (12-61)  (mesh) | Symptomatic recurrences | 8/19 (42) | 3/35 (9) |
|  |  |  |  |  |  |  |  | Anatomical recurrences | 8/19 (42) | 3/35 (9) |
|  |  |  |  |  |  |  |  | Complications  Mesh-associated | NA | NA  1/35 (3)  Esophageal erosion |
|  |  |  |  |  |  |  |  | Reoperations | 4/19 (21) | 1/35 (3) |
| Gouvas et al. [[11](#_ENREF_11)]^‡^ | 2011 | 68 | SEC-SC  PEH  LHH | 360  270 | Circ.  U | PP (12)  Comp. (8) | 12 | Symptomatic recurrences | NA | NA |
|  |  |  |  |  |  |  |  | Anatomical recurrences | 2/16 (13)^§^ | 3/20 (15) |
|  |  |  |  |  |  |  |  | Complications  Mesh-associated | NA | NA  3/20 (15)  Stenoses  1/20 (5) esohageal erosion |
|  |  |  |  |  |  |  |  | Reoperations | 0/48 (0) | 2/20 (10) |
| Schildberg et al. [[12](#_ENREF_12)] | 2012 | 94 | SEC-SC  LHH | 360 | V | PTFE | 60 | Symptomatic recurrences | 6/58 (10) | 2/36 (6) |
|  |  |  |  |  |  |  |  | Anatomical recurrences | 6/58 (10) | 2/36 (6) |
|  |  |  |  |  |  |  |  | Complications  Mesh-associated | 20/58 (34) | 7/36 (19)  0/36 (0) |
|  |  |  |  |  |  |  |  | Reoperations | NA | NA |
| Grubnik et al. [[13](#_ENREF_13)] | 2013 | 234 | SEC-SC  LHH | 360 | V | PP | 29 | Symptomatic recurrences | 11/92 (12) | 7/142 (5) |
|  |  |  |  |  |  |  |  | Anatomical recurrences | 12/92 (13) | 8/142 (6) |
|  |  |  |  |  |  |  |  | Complications  Mesh-associated | NA | NA |
|  |  |  |  |  |  |  |  | Reoperations | 5/92 (5) | 1/142 (1) |
| SC, single center; MC, multi center; PEH, paraesophageal hernia; SEC, case series with control group; CCS, case control study; RCT, randomized controlled trial; LHH, large hiatal hernia; U, U-shape; Circ., circular; V, V-shape; Rect., rectangular; PP, polypropylene; SIS, small intestinal submucosa; DM, dermal matrix; Comp., Composite mesh; PTFE, polytetrafluorethylene; PG, polyglactine; NA, not answered. *Early results. ^†^Late results. ^‡^Not included in the meta-analysis of complications, since none were mentioned. ^§^Only 16 of 48 patients with suture cruroplasty were comparable to the 20 patients with mesh-reinforced cruroplasty.  **References** | | | | | | | | | | |

1. Frantzides CT, Madan AK, Carlson MA, Stavropoulos GP. A prospective, randomized trial of laparoscopic polytetrafluoroethylene (PTFE) patch repair vs simple cruroplasty for large hiatal hernia. Archives of surgery. 2002;137(6):649-52. PubMed PMID: 12049534.

2. Oelschlager BK, Pellegrini CA, Hunter J, Soper N, Brunt M, Sheppard B, et al. Biologic prosthesis reduces recurrence after laparoscopic paraesophageal hernia repair: a multicenter, prospective, randomized trial. Annals of surgery. 2006;244(4):481-90. doi: 10.1097/01.sla.0000237759.42831.03. PubMed PMID: 16998356; PubMed Central PMCID: PMC1856552.

3. Oelschlager BK, Pellegrini CA, Hunter JG, Brunt ML, Soper NJ, Sheppard BC, et al. Biologic prosthesis to prevent recurrence after laparoscopic paraesophageal hernia repair: long-term follow-up from a multicenter, prospective, randomized trial. Journal of the American College of Surgeons. 2011;213(4):461-8. doi: 10.1016/j.jamcollsurg.2011.05.017. PubMed PMID: 21715189.

4. Watson DI, Thompson SK, Devitt PG, Smith L, Woods SD, Aly A, et al. Laparoscopic repair of very large hiatus hernia with sutures versus absorbable mesh versus nonabsorbable mesh: a randomized controlled trial. Annals of surgery. 2015;261(2):282-9. doi: 10.1097/SLA.0000000000000842. PubMed PMID: 25119120.

5. Ringley CD, Bochkarev V, Ahmed SI, Vitamvas ML, Oleynikov D. Laparoscopic hiatal hernia repair with human acellular dermal matrix patch: our initial experience. American journal of surgery. 2006;192(6):767-72. doi: 10.1016/j.amjsurg.2006.08.042. PubMed PMID: 17161091.

6. Hui TT, Thoman DS, Spyrou M, Phillips EH. Mesh crural repair of large paraesophageal hiatal hernias. The American surgeon. 2001;67(12):1170-4. PubMed PMID: 11768823.

7. Leeder PC, Smith G, Dehn TC. Laparoscopic management of large paraesophageal hiatal hernia. Surgical endoscopy. 2003;17(9):1372-5. doi: 10.1007/s00464-002-9192-0. PubMed PMID: 12820060.

8. Muller-Stich BP, Holzinger F, Kapp T, Klaiber C. Laparoscopic hiatal hernia repair: long-term outcome with the focus on the influence of mesh reinforcement. Surgical endoscopy. 2006;20(3):380-4. doi: 10.1007/s00464-004-2272-6. PubMed PMID: 16432659.

9. Morino M, Giaccone C, Pellegrino L, Rebecchi F. Laparoscopic management of giant hiatal hernia: factors influencing long-term outcome. Surgical endoscopy. 2006;20(7):1011-6. doi: 10.1007/s00464-005-0550-6. PubMed PMID: 16763927.

10. Zaninotto G, Portale G, Costantini M, Fiamingo P, Rampado S, Guirroli E, et al. Objective follow-up after laparoscopic repair of large type III hiatal hernia. Assessment of safety and durability. World journal of surgery. 2007;31(11):2177-83. doi: 10.1007/s00268-007-9212-2. PubMed PMID: 17726627.

11. Gouvas N, Tsiaoussis J, Athanasakis E, Zervakis N, Pechlivanides G, Xynos E. Simple suture or prosthesis hiatal closure in laparoscopic repair of paraesophageal hernia: a retrospective cohort study. Diseases of the esophagus : official journal of the International Society for Diseases of the Esophagus / ISDE. 2011;24(2):69-78. doi: 10.1111/j.1442-2050.2010.01094.x. PubMed PMID: 20659144.

12. Schildberg CW, Perrakis A, Croner R, Schellerer V, Haupt W, Weidinger T, et al. [Results of surgical treatment of hiatal hernia]. Zentralblatt fur Chirurgie. 2014;139(1):66-71. doi: 10.1055/s-0032-1315116. PubMed PMID: 23115031.

13. Grubnik VV, Malynovskyy AV. Laparoscopic repair of hiatal hernias: new classification supported by long-term results. Surgical endoscopy. 2013;27(11):4337-46. doi: 10.1007/s00464-013-3069-2. PubMed PMID: 23877759.
