# Supplementary material for: Use of Mesh in Laparoscopic Paraesophageal Hernia Repair: A Meta-Analysis and Risk-Benefit Analysis
Source: PLoS One. 2015 Oct 15;10(10):e0139547. doi: 10.1371/journal.pone.0139547 (PMC4607492; doi:10.1371/journal.pone.0139547)
Supplement: S2 Table — (DOCX) [file pone.0139547.s003.docx]

| **Supporting Information**  **S2 Table.**  Studies included in the systematic review of mesh-associated complications (observational clinical studies) | | | | | | | |
| --- | --- | --- | --- | --- | --- | --- | --- |
| **Study** | **Year** | **N** | **Mesh material [material (n)]** | **Mesh-associated complications (n)** | **Type of mesh-associated complication** | **Clavien grade** | **Symptomatic recurrences in relation to anatomical recurrences [n/m (%)]** |
| Alicuben et al.  [[1](#_ENREF_1)] | 2014 | 82 | Bio | 0 |  |  | NA |
| Alicuben et al.  [[2](#_ENREF_2)] | 2014 | 114 | PGly | 0 |  |  | NA |
| Silecchia et al.  [[3](#_ENREF_3)] | 2014 | 43 | Comp. | 0 |  |  | NA |
| Kang et al.  [[4](#_ENREF_4)] | 2014 | 89 | PTFE | NA |  |  | NA |
| Stiven et al.  [[5](#_ENREF_5)] | 2013 | 114 | PP | NA |  |  | NA |
| Linke et al. [[6](#_ENREF_6)] | 2014 | 55 | PP | 1 | stenosis | II | NA |
| Bjelovic et al. [[7](#_ENREF_7)] | 2014 | 5 | NA | NA |  |  | NA |
| Gebhart et al. [[8](#_ENREF_8)] | 2013 | 92 | PGly | 3 | stenosis | II | NA |
| Lew PS et al. [[9](#_ENREF_9)] | 2013 | 1 | Comp. | NA |  |  | NA |
| Stiven et al. [[5](#_ENREF_5)] | 2013 | 114 | PP | NA |  |  | NA |
| Targarona et al. [[10](#_ENREF_10)] | 2013 | 9 | PTFE (6)  PE (3) | 1 (PTFE)  2 (PTFE)  2 (PTFE) | gastric erosion  stenosis  stenosis | III  III  II | NA |
| Toydemir et al.[[11](#_ENREF_11)] | 2013 | 14 | PP | NA |  |  | NA |
| Grubnik et al. [[12](#_ENREF_12)] | 2013 | 50 | PP | NA |  |  | 1/11 (1) |
| Brandalise et al. [[13](#_ENREF_13)] | 2012 | 70 | PP | 0 |  |  | NA |
| Chilintseva et al. [[14](#_ENREF_14)] | 2012 | 58 | PP (13)  PE (4)  PTFE (40)  Comp. (1) | 2 (PTFE) | stenoses | III | NA |
| Li et al. [[15](#_ENREF_15)] | 2012 | 27 | PTFE (NA)  Bio (NA) | NA |  |  | NA |
| Stavropoulos et al. [[16](#_ENREF_16)] | 2012 | 45 | PTFE | 0 |  |  | NA |
| Diaz et al. [[17](#_ENREF_17)] | 2011 | 46 | Bio | 0 |  |  | NA |
| Goers et al.  [[18](#_ENREF_18)] | 2011 | 56 | Bio | NA |  |  | NA |
| Inaba et al. [[19](#_ENREF_19)] | 2011 | 1 | PTFE | NA |  |  | NA |
| Ma et al. [[20](#_ENREF_20)] | 2011 | 23 | PTFE | 0 |  |  | NA |
| Kanellos et al. [[21](#_ENREF_21)] | 2011 | 26 | PP | 0 |  |  | NA |
| Obeida FW et al. [[22](#_ENREF_22)] | 2011 | 1 | PG | NA |  |  | NA |
| Parsak et al.  [[23](#_ENREF_23)] | 2011 | 150 | PP (75)  PG (75) | 0 |  |  | NA |
| Zehetner et al. [[24](#_ENREF_24)] | 2011 | 61 | Bio (33)  PG (28) | 0 |  |  | NA |
| Koch et al. [[25](#_ENREF_25)] | 2011 | 54 | Comp. | 0 |  |  | 13/15 (87) |
| Mittal et al. [[26](#_ENREF_26)] | 2011 | 10 | PTFE (NA)  Bio (NA) | 0 |  |  | NA |
| Wassenaar et al. [[27](#_ENREF_27)] | 2011 | 126 | Bio | 0 |  |  | NA |
| Braghetto et al. [[28](#_ENREF_28)] | 2010 | 23 | PG (8)  Comp. (13)  Bio (2) | 0 |  |  | 15/15 (100) |
| Luketich et al. [[29](#_ENREF_29)] | 2010 | 88 | NA | 0 |  |  | NA |
| Zehetner et al. [[30](#_ENREF_30)] | 2010 | 35 | PG | 0 |  |  | NA |
| Diez Tabernilla et al. [[31](#_ENREF_31)] | 2009 | 10 | NA | NA |  |  | NA |
| Fortelny et al. [[32](#_ENREF_32)] | 2009 | 1 | PP | 0 |  |  | NA |
| Hazebroek et al. [[33](#_ENREF_33)] | 2009 | 19 | PTFE | 0 |  |  | NA |
| Müller-Stich et al. [[34](#_ENREF_34)] | 2009 | 306 | PP | 3 | stenosis | II | NA |
| Pagan Pomar et al. [[35](#_ENREF_35)] | 2009 | 7 | PTFE (2)  Comp (5) | 0 |  |  | NA |
| Ortiz et al. [[36](#_ENREF_36)] | 2009 | 20 | PTFE | 1 | esophageal erosion | III | NA |
| Soricelli et al. [[37](#_ENREF_37)] | 2009 | 204 | PP | 1 | esophageal erosion | NA | 7/10 (70) |
| Varela et al. [[38](#_ENREF_38)] | 2009 | 5 | Bio | 0 |  |  | NA |
| Zügel et al. [[39](#_ENREF_39)] | 2009 | 9 | Comp (7)  PE (2) | 1 (PE) | erosion of the aorta | III | NA |
| Hazebroek et al. [[40](#_ENREF_40)] | 2008 | 40 | PP | 0 |  |  | 0/1 (0) |
| Fumagalli et al. [[41](#_ENREF_41)] | 2008 | 6 | Bio | 0 |  |  | 0/3 (0) |
| Granderath et al. [[42](#_ENREF_42)] | 2008 | 33 | PP | 0 |  |  | 2/2 (100) |
| Griffith et al. [[43](#_ENREF_43)] | 2008 | 15 | PTFE | 3 | esophageal erosion | II | NA |
| Palanivelu et al. [[44](#_ENREF_44)] | 2008 | 6 | Comp. (2)  PTFE (2)  NA (2) | NA |  |  | NA |
| Müller-Stich et al. [[45](#_ENREF_45)] | 2008 | 22 | PP | 0 |  |  | 2/8 (25) |
| Varela et al. [[46](#_ENREF_46)] | 2008 | 5 | PE | 0 |  |  | NA |
| Linke et al. [[45](#_ENREF_45)] | 2008 | 48 | PP | NA |  |  | NA |
| Lee et al. [[47](#_ENREF_47)] | 2008 | 52 | DM | 0 |  |  | NA |
| Boushey et al. [[48](#_ENREF_48)] | 2008 | 2 | PTFE | NA |  |  | 2/5 (40) |
| Gelmini et al. [[49](#_ENREF_49)] | 2007 | 1 | PTFE | 0 |  |  | NA |
| Granderath et al. [[50](#_ENREF_50)] | 2007 | 23 | PP (12)  Comp. (5)  PTFE (6) | 0 |  |  | NA |
| Jacobs et al. [[51](#_ENREF_51)] | 2007 | 127 | Bio | NA |  |  | NA |
| Lee et al. [[52](#_ENREF_52)] | 2007 | 17 | Bio | 1 | gastric erosion | II | 1/2 (50) |
| Lubezky et al. [[53](#_ENREF_53)] | 2007 | 59 | PTFE (NA) Comp (NA) | 0 |  |  | 9/15 (60) |
| Turkcaparet al. [[54](#_ENREF_54)] | 2007 | 176 | PP | 0 |  |  | NA |
| Draaisma et al. [[55](#_ENREF_55)] | 2006 | 1 | PTFE | NA |  |  | NA |
| Gangopadhyay et al. [[56](#_ENREF_56)] | 2006 | 31 | Bio (29)  PTFE (2) | NA |  |  | NA |
| Granderath et al. [[57](#_ENREF_57)] | 2006 | 20 | PP | NA |  |  | NA |
| Parameswaran et al. [[58](#_ENREF_58)] | 2006 | 17 | PP (10)  PTFE (1)  Bio (6) | NA |  |  | 2/4 (50) |
| Wisbach et al. [[59](#_ENREF_59)] | 2006 | 11 | Bio | 1 | stenosis | II | 0/1 (0) |
| Zilberstein et al. [[60](#_ENREF_60)] | 2005 | 7 | PE | 1 | esophageal erosion | II | NA |
| Casaccia et al. [[61](#_ENREF_61)] | 2005 | 27 | Comp. | 0 |  |  | NA |
| Granderath et al. [[62](#_ENREF_62)] | 2005 | 50 | PP | 0 |  |  | NA |
| Gryska et al. [[63](#_ENREF_63)] | 2005 | 135 | PTFE | 0 |  |  | NA |
| Johnson et al. [[64](#_ENREF_64)] | 2005 | 3 | Bio | 0 |  |  | NA |
| Dally et al. [[65](#_ENREF_65)] | 2004 | 1175 | PTFE | 11 | gastric erosion (5)  gastroesophageal erosion (3)  esophageal erosion (3) | NA | NA |
| Horstmann et al. [[66](#_ENREF_66)] | 2004 | 16 | PP | NA |  |  | NA |
| Diaz et al. [[67](#_ENREF_67)] | 2003 | 9 | PP | NA |  |  | 13/21 (62) |
| Strange et al. [[68](#_ENREF_68)] | 2003 | 12 | Bio | NA |  |  | NA |
| Granderath et al. [[69](#_ENREF_69)] | 2003 | 24 | PP | 0 |  |  | NA |
| Oelschlager et al. [[70](#_ENREF_70)] | 2003 | 9 | Bio | 0 |  |  | 0/1 (0) |
| Keidar et al. [[71](#_ENREF_71)] | 2003 | 10 | PTFE (6)  PP (4) | 0 |  |  | NA |
| Champion et al. [[72](#_ENREF_72)] | 2003 | 52 | PP | 0 |  |  | NA |
| Casaccia et al. [[73](#_ENREF_73)] | 2002 | 8 | PTFE | NA |  |  | NA |
| Granderath et al. [[74](#_ENREF_74)] | 2002 | 170 | PP | 0 |  |  | 17/22 (77) |
| Kamolz et al. [[74](#_ENREF_74)] | 2002 | 100 | PP | 0 |  |  | 4/9 (44) |
| Livingston et al. [[75](#_ENREF_75)] | 2001 | 10 | Comp | 0 |  |  | NA |
| Athanasakis et al. [[76](#_ENREF_76)] | 2001 | 3 | PTFE | NA |  |  | NA |
| Basso et al. [[77](#_ENREF_77)] | 2000 | 65 | PP | 0 |  |  | NA |
| Luketich et al. [[78](#_ENREF_78)] | 2000 | 4 | PTFE | NA |  |  | NA |
| Basso et al. [[79](#_ENREF_79)] | 1999 | 12 | PP | 0 |  |  | NA |
| Wu et al. [[80](#_ENREF_80)] | 1999 | 5 | PP | 0 |  |  | 3/8 (38) |
| Bärlehner et al. [[81](#_ENREF_81)] | 1998 | 5 | PP | NA |  |  | NA |
| Medina et al. [[82](#_ENREF_82)] | 1998 | 2 | PTFE (1)  PP (1) | NA |  |  | NA |
| Hawasli et al. [[83](#_ENREF_83)] | 1998 | 27 | PP | NA |  |  | NA |
| Frantzides et al. [[84](#_ENREF_84)] | 1997 | 3 | PTFE | 0 |  |  | NA |
| Huntington et al. [[85](#_ENREF_85)] | 1997 | 8 | PP | 0 |  |  | NA |
| Paul et al. [[86](#_ENREF_86)] | 1997 | 3 | PTFE | NA |  |  | NA |
| Watanabe et al. [[87](#_ENREF_87)] | 1997 | 1 | PP | 0 |  |  | NA |
| Behrns et al. [[88](#_ENREF_88)] | 1996 | 2 | NA | NA |  |  | 0/1 (0) |
| Edelman et al. [[89](#_ENREF_89)] | 1995 | 5 | PP | 1 | stenosis | NA | NA |
| Kuster et al. [[90](#_ENREF_90)] | 1993 | 5 | PE | NA |  |  | NA |

PP; polypropylene; PGly, polyglycolide; Bio; biomesh (including small intestinal submucosa and dermal matrix); Comp., composite mesh; PTFE, polytetrafluorethylene; PE, polyethylene; PG, polyglactine; NA, not answered.

**References**

1. Alicuben ET, Worrell SG, DeMeester SR. Impact of crural relaxing incisions, Collis gastroplasty, and non-cross-linked human dermal mesh crural reinforcement on early hiatal hernia recurrence rates. Journal of the American College of Surgeons. 2014;219(5):988-92. doi: 10.1016/j.jamcollsurg.2014.07.937. PubMed PMID: 25256373.

2. Alicuben ET, Worrell SG, DeMeester SR. Resorbable biosynthetic mesh for crural reinforcement during hiatal hernia repair. The American surgeon. 2014;80(10):1030-3. PubMed PMID: 25264654.

3. Silecchia G, Iossa A, Cavallaro G, Rizzello M, Longo F. Reinforcement of hiatal defect repair with absorbable mesh fixed with non-permanent devices. Minimally invasive therapy & allied technologies : MITAT : official journal of the Society for Minimally Invasive Therapy. 2014;23(5):302-8. doi: 10.3109/13645706.2014.909853. PubMed PMID: 24773371.

4. Kang T, Urrego H, Gridley A, Richardson WS. Pledgeted repair of giant hiatal hernia provides excellent long-term results. Journal of laparoendoscopic & advanced surgical techniques Part A. 2014;24(10):684-7. doi: 10.1089/lap.2013.0447. PubMed PMID: 25181468.

5. Stiven PN, Hansen R, Richardson A, Leibman S, Smith GS. Postoperative Dysphagia in laparoscopic paraesophageal hernia repair: the effect of distal esophageal angulation. Surgical laparoscopy, endoscopy & percutaneous techniques. 2013;23(5):449-52. doi: 10.1097/SLE.0b013e31829ce60a. PubMed PMID: 24105284.

6. Linke GR, Gehrig T, Hogg LV, Gohl A, Kenngott H, Schafer F, et al. Laparoscopic mesh-augmented hiatoplasty without fundoplication as a method to treat large hiatal hernias. Surgery today. 2014;44(5):820-6. doi: 10.1007/s00595-013-0609-2. PubMed PMID: 23670038; PubMed Central PMCID: PMC3986894.

7. Bjelovic M, Babic T, Gunjic D, Veselinovic M, Spica B. Laparoscopic repair of hiatal hernias: experience after 200 consecutive cases. Srpski arhiv za celokupno lekarstvo. 2014;142(7-8):424-30. PubMed PMID: 25233686.

8. Gebhart A, Vu S, Armstrong C, Smith BR, Nguyen NT. Initial outcomes of laparoscopic paraesophageal hiatal hernia repair with mesh. The American surgeon. 2013;79(10):1017-21. PubMed PMID: 24160791.

9. Lew PS, Wong AS. Laparoscopic mesh repair of parahiatal hernia: a case report. Asian journal of endoscopic surgery. 2013;6(3):231-3. doi: 10.1111/ases.12039. PubMed PMID: 23879418.

10. Targarona EM, Grisales S, Uyanik O, Balague C, Pernas JC, Trias M. Long-term outcome and quality of life after laparoscopic treatment of large paraesophageal hernia. World journal of surgery. 2013;37(8):1878-82. doi: 10.1007/s00268-013-2047-0. PubMed PMID: 23604303.

11. Toydemir T, Cipe G, Karatepe O, Yerdel MA. Laparoscopic management of totally intra-thoracic stomach with chronic volvulus. World journal of gastroenterology : WJG. 2013;19(35):5848-54. doi: 10.3748/wjg.v19.i35.5848. PubMed PMID: 24124329; PubMed Central PMCID: PMC3793138.

12. Grubnik VV, Malynovskyy AV. Laparoscopic repair of hiatal hernias: new classification supported by long-term results. Surgical endoscopy. 2013;27(11):4337-46. doi: 10.1007/s00464-013-3069-2. PubMed PMID: 23877759.

13. Brandalise A, Aranha NC, Brandalise NA. The polypropylene mesh in the laparoscopic repair of large hiatal hernias: technical aspects. Arquivos brasileiros de cirurgia digestiva : ABCD = Brazilian archives of digestive surgery. 2012;25(4):224-8. PubMed PMID: 23411919.

14. Chilintseva N, Brigand C, Meyer C, Rohr S. Laparoscopic prosthetic hiatal reinforcement for large hiatal hernia repair. Journal of visceral surgery. 2012;149(3):e215-20. doi: 10.1016/j.jviscsurg.2012.01.006. PubMed PMID: 22364855.

15. Li J, Rosenthal RJ, Roy M, Szomstein S, Sesto M. Experience of laparoscopic paraesophageal hernia repair at a single institution. American journal of surgery. 2012;204(1):60-5. doi: 10.1016/j.amjsurg.2011.06.026. PubMed PMID: 21992807.

16. Stavropoulos G, Flessas, II, Mariolis-Sapsakos T, Zagouri F, Theodoropoulos G, Toutouzas K, et al. Laparoscopic repair of giant paraesophageal hernia with synthetic mesh: 45 consecutive cases. The American surgeon. 2012;78(4):432-5. PubMed PMID: 22472400.

17. Diaz DF, Roth JS. Laparoscopic paraesophageal hernia repair with acellular dermal matrix cruroplasty. JSLS : Journal of the Society of Laparoendoscopic Surgeons / Society of Laparoendoscopic Surgeons. 2011;15(3):355-60. doi: 10.4293/108680811X13125733356594. PubMed PMID: 21985724; PubMed Central PMCID: PMC3183546.

18. Goers TA, Cassera MA, Dunst CM, Swanstrom LL. Paraesophageal hernia repair with biomesh does not increase postoperative dysphagia. Journal of gastrointestinal surgery : official journal of the Society for Surgery of the Alimentary Tract. 2011;15(10):1743-9. doi: 10.1007/s11605-011-1596-5. PubMed PMID: 21773871.

19. Inaba K, Sakurai Y, Isogaki J, Komori Y, Uyama I. Laparoscopic repair of hiatal hernia with mesenterioaxial volvulus of the stomach. World journal of gastroenterology : WJG. 2011;17(15):2054-7. doi: 10.3748/wjg.v17.i15.2054. PubMed PMID: 21528087; PubMed Central PMCID: PMC3082762.

20. Ma B, Tian W, Chen L, Liu P. Laparoscopic repair of esophageal hiatal hernia. Journal of Huazhong University of Science and Technology Medical sciences = Hua zhong ke ji da xue xue bao Yi xue Ying De wen ban = Huazhong keji daxue xuebao Yixue Yingdewen ban. 2011;31(2):231-4. doi: 10.1007/s11596-011-0258-x. PubMed PMID: 21505991.

21. Kanellos D, Moesta KT, Schug-Pass C, Kockerling F. [Hiatoplasty reinforcement by means of a lightweight titanized polypropylene mesh fixed with fibrin glue]. Zentralblatt fur Chirurgie. 2011;136(3):244-8. doi: 10.1055/s-0030-1247261. PubMed PMID: 20309806.

22. Obeidat FW, Lang RA, Knauf A, Thomas MN, Huttl TK, Zugel NP, et al. Laparoscopic anterior hemifundoplication and hiatoplasty for the treatment of upside-down stomach: mid- and long-term results after 40 patients. Surgical endoscopy. 2011;25(7):2230-5. doi: 10.1007/s00464-010-1537-5. PubMed PMID: 21359905.

23. Parsak CK, Erel S, Seydaoglu G, Akcam T, Sakman G. Laparoscopic antireflux surgery with polyglactin (vicryl) mesh. Surgical laparoscopy, endoscopy & percutaneous techniques. 2011;21(6):443-9. doi: 10.1097/SLE.0b013e31823acc87. PubMed PMID: 22146169.

24. Zehetner J, Demeester SR, Ayazi S, Kilday P, Augustin F, Hagen JA, et al. Laparoscopic versus open repair of paraesophageal hernia: the second decade. Journal of the American College of Surgeons. 2011;212(5):813-20. doi: 10.1016/j.jamcollsurg.2011.01.060. PubMed PMID: 21435915.

25. Koch OO, Asche KU, Berger J, Weber E, Granderath FA, Pointner R. Influence of the size of the hiatus on the rate of reherniation after laparoscopic fundoplication and refundopilication with mesh hiatoplasty. Surgical endoscopy. 2011;25(4):1024-30. doi: 10.1007/s00464-010-1308-3. PubMed PMID: 20734068.

26. Mittal SK, Bikhchandani J, Gurney O, Yano F, Lee T. Outcomes after repair of the intrathoracic stomach: objective follow-up of up to 5 years. Surgical endoscopy. 2011;25(2):556-66. doi: 10.1007/s00464-010-1219-3. PubMed PMID: 20623236.

27. Wassenaar EB, Mier F, Sinan H, Petersen RP, Martin AV, Pellegrini CA, et al. The safety of biologic mesh for laparoscopic repair of large, complicated hiatal hernia. Surgical endoscopy. 2012;26(5):1390-6. doi: 10.1007/s00464-011-2045-y. PubMed PMID: 22083339.

28. Braghetto I, Korn O, Csendes A, Burdiles P, Valladares H, Brunet L. Postoperative results after laparoscopic approach for treatment of large hiatal hernias: is mesh always needed? Is the addition of an antireflux procedure necessary? International surgery. 2010;95(1):80-7. PubMed PMID: 20480847.

29. Luketich JD, Nason KS, Christie NA, Pennathur A, Jobe BA, Landreneau RJ, et al. Outcomes after a decade of laparoscopic giant paraesophageal hernia repair. The Journal of thoracic and cardiovascular surgery. 2010;139(2):395-404, e1. doi: 10.1016/j.jtcvs.2009.10.005. PubMed PMID: 20004917; PubMed Central PMCID: PMC2813424.

30. Zehetner J, Lipham JC, Ayazi S, Oezcelik A, Abate E, Chen W, et al. A simplified technique for intrathoracic stomach repair: laparoscopic fundoplication with Vicryl mesh and BioGlue crural reinforcement. Surgical endoscopy. 2010;24(3):675-9. doi: 10.1007/s00464-009-0662-5. PubMed PMID: 19690911.

31. Diez Tabernilla M, Ruiz-Tovar J, Grajal Marino R, Calero Garcia P, Pina Hernandez JD, Chames Vaisman A, et al. Paraesophageal hiatal hernia. Open vs. laparoscopic surgery. Revista espanola de enfermedades digestivas : organo oficial de la Sociedad Espanola de Patologia Digestiva. 2009;101(10):706-11. PubMed PMID: 19899938.

32. Fortelny RH, Petter-Puchner AH, Glaser KS. Fibrin sealant (Tissucol) for the fixation of hiatal mesh in the repair of giant paraesophageal hernia: a case report. Surgical laparoscopy, endoscopy & percutaneous techniques. 2009;19(3):e91-4. doi: 10.1097/SLE.0b013e31819f2066. PubMed PMID: 19542837.

33. Hazebroek EJ, Koak Y, Berry H, Leibman S, Smith GS. Critical evaluation of a novel DualMesh repair for large hiatal hernias. Surgical endoscopy. 2009;23(1):193-6. doi: 10.1007/s00464-008-9772-8. PubMed PMID: 18320282.

34. Muller-Stich BP, Koninger J, Muller-Stich BH, Schafer F, Warschkow R, Mehrabi A, et al. Laparoscopic mesh-augmented hiatoplasty as a method to treat gastroesophageal reflux without fundoplication: single-center experience with 306 consecutive patients. American journal of surgery. 2009;198(1):17-24. doi: 10.1016/j.amjsurg.2008.07.050. PubMed PMID: 19178902.

35. Pagan Pomar A, Palma Zamora E, Ochogavia Segui A, Llabres Rosello M. Laparoscopic surgery into mixed hiatal hernia. Results pre-operative and post-operative. Revista espanola de enfermedades digestivas : organo oficial de la Sociedad Espanola de Patologia Digestiva. 2009;101(9):623-30. PubMed PMID: 19803665.

36. Ortiz I, Targarona EM, Pallares L, Marinello F, Balague C, Trias M. [Quality of life and long-term results of reinterventions performed by laparoscopy after oesophageal hiatus surgery]. Cirugia espanola. 2009;86(2):72-8. doi: 10.1016/j.ciresp.2009.02.005. PubMed PMID: 19497566.

37. Soricelli E, Basso N, Genco A, Cipriano M. Long-term results of hiatal hernia mesh repair and antireflux laparoscopic surgery. Surgical endoscopy. 2009;23(11):2499-504. doi: 10.1007/s00464-009-0425-3. PubMed PMID: 19343437.

38. Varela JE, Jacks SP. Laparoscopic circular biomesh hiatoplasty during paraesophageal hernia repair. Surgical innovation. 2009;16(2):124-8. doi: 10.1177/1553350609336420. PubMed PMID: 19443865.

39. Zugel N, Lang RA, Kox M, Huttl TP. Severe complication of laparoscopic mesh hiatoplasty for paraesophageal hernia. Surgical endoscopy. 2009;23(11):2563-7. doi: 10.1007/s00464-009-0456-9. PubMed PMID: 19440795.

40. Hazebroek EJ, Ng A, Yong DH, Berry H, Leibman S, Smith GS. Clinical evaluation of laparoscopic repair of large hiatal hernias with TiMesh. ANZ journal of surgery. 2008;78(10):914-7. doi: 10.1111/j.1445-2197.2008.04691.x. PubMed PMID: 18959648.

41. Fumagalli U, Bona S, Caputo M, Elmore U, Battafarano F, Pestalozza A, et al. Are Surgisis biomeshes effective in reducing recurrences after laparoscopic repair of large hiatal hernias? Surgical laparoscopy, endoscopy & percutaneous techniques. 2008;18(5):433-6. doi: 10.1097/SLE.0b013e3181802ca7. PubMed PMID: 18936659.

42. Granderath FA, Granderath UM, Pointner R. Laparoscopic revisional fundoplication with circular hiatal mesh prosthesis: the long-term results. World journal of surgery. 2008;32(6):999-1007. doi: 10.1007/s00268-008-9558-0. PubMed PMID: 18373118.

43. Griffith PS, Valenti V, Qurashi K, Martinez-Isla A. Rejection of goretex mesh used in prosthetic cruroplasty: a case series. International journal of surgery. 2008;6(2):106-9. doi: 10.1016/j.ijsu.2007.12.004. PubMed PMID: 18234570.

44. Palanivelu C, Rangarajan M, Jategaonkar PA, Parthasarathi R, Balu K. Laparoscopic repair of parahiatal hernias with mesh: a retrospective study. Hernia : the journal of hernias and abdominal wall surgery. 2008;12(5):521-5. doi: 10.1007/s10029-008-0380-2. PubMed PMID: 18661099.

45. Muller-Stich BP, Linke GR, Borovicka J, Marra F, Warschkow R, Lange J, et al. Laparoscopic mesh-augmented hiatoplasty as a treatment of gastroesophageal reflux disease and hiatal hernias-preliminary clinical and functional results of a prospective case series. American journal of surgery. 2008;195(6):749-56. doi: 10.1016/j.amjsurg.2007.06.022. PubMed PMID: 18353273.

46. Varela E, Hinojosa M, Nguyen NT. Polyester composite mesh for laparoscopic paraesophageal hernia repair. Surgical innovation. 2008;15(2):90-4. doi: 10.1177/1553350608318143. PubMed PMID: 18448446.

47. Lee YK, James E, Bochkarev V, Vitamvas M, Oleynikov D. Long-term outcome of cruroplasty reinforcement with human acellular dermal matrix in large paraesophageal hiatal hernia. Journal of gastrointestinal surgery : official journal of the Society for Surgery of the Alimentary Tract. 2008;12(5):811-5. doi: 10.1007/s11605-007-0463-x. PubMed PMID: 18181005.

48. Boushey RP, Moloo H, Burpee S, Schlachta CM, Poulin EC, Haggar F, et al. Laparoscopic repair of paraesophageal hernias: a Canadian experience. Canadian journal of surgery Journal canadien de chirurgie. 2008;51(5):355-60. PubMed PMID: 18841230; PubMed Central PMCID: PMC2556541.

49. Gelmini R, Andreotti A, Saviano M. Laparoscopic treatment of paraesophageal and mixed diaphragmatic hernias. Report of two cases. Minerva chirurgica. 2007;62(3):191-6. PubMed PMID: 17519845.

50. Granderath FA, Schweiger UM, Pointner R. Laparoscopic antireflux surgery: tailoring the hiatal closure to the size of hiatal surface area. Surgical endoscopy. 2007;21(4):542-8. doi: 10.1007/s00464-006-9041-7. PubMed PMID: 17103275.

51. Jacobs M, Gomez E, Plasencia G, Lopez-Penalver C, Lujan H, Velarde D, et al. Use of surgisis mesh in laparoscopic repair of hiatal hernias. Surgical laparoscopy, endoscopy & percutaneous techniques. 2007;17(5):365-8. doi: 10.1097/SLE.0b013e318123fc49. PubMed PMID: 18049393.

52. Lee E, Frisella MM, Matthews BD, Brunt LM. Evaluation of acellular human dermis reinforcement of the crural closure in patients with difficult hiatal hernias. Surgical endoscopy. 2007;21(4):641-5. doi: 10.1007/s00464-006-9117-4. PubMed PMID: 17287920.

53. Lubezky N, Sagie B, Keidar A, Szold A. Prosthetic mesh repair of large and recurrent diaphragmatic hernias. Surgical endoscopy. 2007;21(5):737-41. doi: 10.1007/s00464-007-9208-x. PubMed PMID: 17458615.

54. Turkcapar A, Kepenekci I, Mahmoud H, Tuzuner A. Laparoscopic fundoplication with prosthetic hiatal closure. World journal of surgery. 2007;31(11):2169-76. doi: 10.1007/s00268-007-9066-7. PubMed PMID: 17610010.

55. Draaisma WA, Simmermacher RK, Broeders IA. Recurrent paraesophageal hernia due to diaphragm rupture: a case report. Hernia : the journal of hernias and abdominal wall surgery. 2006;10(3):282-5. doi: 10.1007/s10029-006-0069-3. PubMed PMID: 16453074.

56. Gangopadhyay N, Perrone JM, Soper NJ, Matthews BD, Eagon JC, Klingensmith ME, et al. Outcomes of laparoscopic paraesophageal hernia repair in elderly and high-risk patients. Surgery. 2006;140(4):491-8; discussion 8-9. doi: 10.1016/j.surg.2006.07.001. PubMed PMID: 17011895.

57. Granderath FA, Kamolz T, Schweiger UM, Pointner R. Impact of laparoscopic nissen fundoplication with prosthetic hiatal closure on esophageal body motility: Results of a prospective randomized trial. Archives of surgery. 2006;141(7):625-32. doi: 10.1001/archsurg.141.7.625. PubMed PMID: 16847231.

58. Parameswaran R, Ali A, Velmurugan S, Adjepong SE, Sigurdsson A. Laparoscopic repair of large paraesophageal hiatus hernia: quality of life and durability. Surgical endoscopy. 2006;20(8):1221-4. doi: 10.1007/s00464-005-0691-7. PubMed PMID: 16865618.

59. Wisbach G, Peterson T, Thoman D. Early results of the use of acellular dermal allograft in type III paraesophageal hernia repair. JSLS : Journal of the Society of Laparoendoscopic Surgeons / Society of Laparoendoscopic Surgeons. 2006;10(2):184-7. PubMed PMID: 16882417; PubMed Central PMCID: PMC3016123.

60. Zilberstein B, Eshkenazy R, Pajecki D, Granja C, Brito AC. Laparoscopic mesh repair antireflux surgery for treatment of large hiatal hernia. Diseases of the esophagus : official journal of the International Society for Diseases of the Esophagus / ISDE. 2005;18(3):166-9. doi: 10.1111/j.1442-2050.2005.00494.x. PubMed PMID: 16045578.

61. Casaccia M, Torelli P, Panaro F, Cavaliere D, Saltalamacchia L, Troilo BM, et al. Laparoscopic tension-free repair of large paraesophageal hiatal hernias with a composite A-shaped mesh: two-year follow-up. Journal of laparoendoscopic & advanced surgical techniques Part A. 2005;15(3):279-84. doi: 10.1089/lap.2005.15.279. PubMed PMID: 15954829.

62. Granderath FA, Schweiger UM, Kamolz T, Asche KU, Pointner R. Laparoscopic Nissen fundoplication with prosthetic hiatal closure reduces postoperative intrathoracic wrap herniation: preliminary results of a prospective randomized functional and clinical study. Archives of surgery. 2005;140(1):40-8. doi: 10.1001/archsurg.140.1.40. PubMed PMID: 15655204.

63. Gryska PV, Vernon JK. Tension-free repair of hiatal hernia during laparoscopic fundoplication: a ten-year experience. Hernia : the journal of hernias and abdominal wall surgery. 2005;9(2):150-5. doi: 10.1007/s10029-004-0312-8. PubMed PMID: 15723153.

64. Johnson JM, Carmody BJ, Jamal MK, DeMaria EJ. Onlay hiatal reinforcement utilizing human acellular dermal matrix: three case series. Surgical innovation. 2005;12(3):239-41. PubMed PMID: 16224645.

65. Dally E, Falk GL. Teflon pledget reinforced fundoplication causes symptomatic gastric and esophageal lumenal penetration. American journal of surgery. 2004;187(2):226-9. doi: 10.1016/j.amjsurg.2003.11.028. PubMed PMID: 14769309.

66. Horstmann R, Klotz A, Classen C, Palmes D. Feasibility of surgical technique and evaluation of postoperative quality of life after laparoscopic treatment of intrathoracic stomach. Langenbeck's archives of surgery / Deutsche Gesellschaft fur Chirurgie. 2004;389(1):23-31. doi: 10.1007/s00423-003-0437-8. PubMed PMID: 14625776.

67. Diaz S, Brunt LM, Klingensmith ME, Frisella PM, Soper NJ. Laparoscopic paraesophageal hernia repair, a challenging operation: medium-term outcome of 116 patients. Journal of gastrointestinal surgery : official journal of the Society for Surgery of the Alimentary Tract. 2003;7(1):59-66; discussion -7. PubMed PMID: 12559186.

68. Strange PS. Small intestinal submucosa for laparoscopic repair of large paraesophageal hiatal hernias: a preliminary report. Surgical technology international. 2003;11:141-3. PubMed PMID: 12931295.

69. Granderath FA, Kamolz T, Schweiger UM, Pointner R. Laparoscopic refundoplication with prosthetic hiatal closure for recurrent hiatal hernia after primary failed antireflux surgery. Archives of surgery. 2003;138(8):902-7. doi: 10.1001/archsurg.138.8.902. PubMed PMID: 12912751.

70. Oelschlager BK, Barreca M, Chang L, Pellegrini CA. The use of small intestine submucosa in the repair of paraesophageal hernias: initial observations of a new technique. American journal of surgery. 2003;186(1):4-8. PubMed PMID: 12842738.

71. Keidar A, Szold A. Laparoscopic repair of paraesophageal hernia with selective use of mesh. Surgical laparoscopy, endoscopy & percutaneous techniques. 2003;13(3):149-54. PubMed PMID: 12819496.

72. Champion JK, Rock D. Laparoscopic mesh cruroplasty for large paraesophageal hernias. Surgical endoscopy. 2003;17(4):551-3. doi: 10.1007/s00464-002-8817-7. PubMed PMID: 12582773.

73. Casaccia M, Torelli P, Panaro F, Cavaliere D, Ventura A, Valente U. Laparoscopic physiological hiatoplasty for hiatal hernia: new composite "A"-shaped mesh. Physical and geometrical analysis and preliminary clinical results. Surgical endoscopy. 2002;16(10):1441-5. doi: 10.1007/s00464-002-9029-x. PubMed PMID: 12085149.

74. Kamolz T, Granderath FA, Bammer T, Pasiut M, Pointner R. Dysphagia and quality of life after laparoscopic Nissen fundoplication in patients with and without prosthetic reinforcement of the hiatal crura. Surgical endoscopy. 2002;16(4):572-7. doi: 10.1007/s00464-001-9136-0. PubMed PMID: 11972190.

75. Livingston CD, Jones HL, Jr., Askew RE, Jr., Victor BE, Askew RE, Sr. Laparoscopic hiatal hernia repair in patients with poor esophageal motility or paraesophageal herniation. The American surgeon. 2001;67(10):987-91. PubMed PMID: 11603559.

76. Athanasakis H, Tzortzinis A, Tsiaoussis J, Vassilakis JS, Xynos E. Laparoscopic repair of paraesophageal hernia. Endoscopy. 2001;33(7):590-4. doi: 10.1055/s-2001-15306. PubMed PMID: 11473330.

77. Basso N, De Leo A, Genco A, Rosato P, Rea S, Spaziani E, et al. 360 degrees laparoscopic fundoplication with tension-free hiatoplasty in the treatment of symptomatic gastroesophageal reflux disease. Surgical endoscopy. 2000;14(2):164-9. PubMed PMID: 10656953.

78. Luketich JD, Raja S, Fernando HC, Campbell W, Christie NA, Buenaventura PO, et al. Laparoscopic repair of giant paraesophageal hernia: 100 consecutive cases. Annals of surgery. 2000;232(4):608-18. PubMed PMID: 10998659; PubMed Central PMCID: PMC1421193.

79. Basso N, Rosato P, De Leo A, Genco A, Rea S, Neri T. "Tension-free" hiatoplasty, gastrophrenic anchorage, and 360 degrees fundoplication in the laparoscopic treatment of paraesophageal hernia. Surgical laparoscopy, endoscopy & percutaneous techniques. 1999;9(4):257-62. PubMed PMID: 10871172.

80. Wu JS, Dunnegan DL, Soper NJ. Clinical and radiologic assessment of laparoscopic paraesophageal hernia repair. Surgical endoscopy. 1999;13(5):497-502. PubMed PMID: 10227951.

81. Barlehner E, Heukrodt B. [Laparoscopic repair of large hiatal hernias with polypropylene mesh]. Zentralblatt fur Chirurgie. 1998;123(11):1303-5. PubMed PMID: 9880852.

82. Medina L, Peetz M, Ratzer E, Fenoglio M. Laparoscopic paraesophageal hernia repair. JSLS : Journal of the Society of Laparoendoscopic Surgeons / Society of Laparoendoscopic Surgeons. 1998;2(3):269-72. PubMed PMID: 9876752; PubMed Central PMCID: PMC3015312.

83. Hawasli A, Zonca S. Laparoscopic repair of paraesophageal hiatal hernia. The American surgeon. 1998;64(8):703-10. PubMed PMID: 9697897.

84. Frantzides CT, Carlson MA. Prosthetic reinforcement of posterior cruroplasty during laparoscopic hiatal herniorrhaphy. Surgical endoscopy. 1997;11(7):769-71. PubMed PMID: 9214330.

85. Huntington TR. Laparoscopic mesh repair of the esophageal hiatus. Journal of the American College of Surgeons. 1997;184(4):399-400. PubMed PMID: 9100687.

86. Paul MG, DeRosa RP, Petrucci PE, Palmer ML, Danovitch SH. Laparoscopic tension-free repair of large paraesophageal hernias. Surgical endoscopy. 1997;11(3):303-7. PubMed PMID: 9079617.

87. Watanabe G, Tanaka J, Odashima S, Kitamura M, Koyama K. Laparoscopic repair of a paraesophageal hiatus hernia without fundoplication. Surgery today. 1997;27(11):1093-6. PubMed PMID: 9413069.

88. Behrns KE, Schlinkert RT. Laparoscopic management of paraesophageal hernia: early results. Journal of laparoendoscopic surgery. 1996;6(5):311-7. PubMed PMID: 8897241.

89. Edelman DS. Laparoscopic paraesophageal hernia repair with mesh. Surgical laparoscopy & endoscopy. 1995;5(1):32-7. PubMed PMID: 7735538.

90. Kuster GG, Gilroy S. Laparoscopic technique for repair of paraesophageal hiatal hernias. Journal of laparoendoscopic surgery. 1993;3(4):331-8. PubMed PMID: 8268502.
