# Supplementary material for: Use of Mesh in Laparoscopic Paraesophageal Hernia Repair: A Meta-Analysis and Risk-Benefit Analysis
Source: PLoS One. 2015 Oct 15;10(10):e0139547. doi: 10.1371/journal.pone.0139547 (PMC4607492; doi:10.1371/journal.pone.0139547)
Supplement: S3 Table — (DOCX) [file pone.0139547.s004.docx]

**Supporting Information**

**S3 Table.** Studies included in the systematic review of mesh-associated complications (case reports)

| **Study** | **Year** | **N** | **Mesh material [material (n)]** | **Mesh-associated complications [complication (n)]** | **Clavien grade** |
| --- | --- | --- | --- | --- | --- |
| Acin-Gandara et al. [1] | 2014 | 1 | PTFE | gastric erosion | II |
| Cano-Valderrama et al. [2] | 2013 | 1 | Comp. | aortic injury | II |
| De Moor et al. [3] | 2012 | 1  2 | Comp. | esophageal erosion  gastric erosion | III  II |
| Makarewicz et al. [4] | 2012 | 1 | Comp. | cardiac tamponade | III |
| Rodriguez-Lago et al. [5] | 2012 | 1 | PP | esophageal erosion | II |
| Paz et al. [6] | 2011 | 1 | DM | cardiac tamponade | II |
| Carpelan-Holmström et al. [7] | 2011 | 1 | PTFE | gastric erosion | II |
| Frantzides et al. [8] | 2012 | 2 | NA | fatal cardiac tamponade (2) | III |
| Hazebroek et al. [9] | 2009 | 1 | PTFE | esophageal erosion | II |
| Kepenekci et al. [10] | 2009 | 1 | NA | esophageal erosion | III |
| Stadlhuber et al. [11] | 2009 | 28 | PP(8)  PTFE (12)  Bio (7)  Comp. (1) | erosion (17)  stenosis (6)  fibrosis (5) | II  [(stenosis (5), erosion (8), fibrosis (5)]  III  [stenosis (1), 9 erosion (9)] |
| Tatum et al. [12] | 2008 | 2 | PTFE | gastric erosion  stenosis | III  II |
| Bonavina et al. [13] | 2007 | 1 | Comp. | stenosis | II |
| Hergueta-Delgado et al. [14] | 2006 | 1 | PTFE | gastric erosion | NA |
| Müller-Stich et al. [15] | 2006 | 1 | PP | fatal cardiac tamponade | III |
| Thijssens et al. [16] | 2002 | 1 | PE | cardiac tamponade | II |
| Baladas et al. [17] | 2000 | 1 | PTFE | gastroesophageal fistula | II |
| Kemppainen et al. [18] | 2000 | 1 | PTFE | fatal cardiac tamponade | III |
| Farlo et al. [19] | 1998 | 1 | NA | cardiac tamponade | NA |

PP; polypropylene; Bio; biomesh (including small intestinal submucosa and dermal matrix); Comp., composite mesh; PTFE, polytetrafluorethylene; PE, polyethylene; NA, not answered.

**References**

1. Acin-Gandara D, Miliani-Molina C, Carneros-Martin J, Martinez-Pineiro J, Vega MD, Pereira-Perez F. Transmural gastric migration of dual-sided PTFE/ePTFEE mesh after laparoscopic surgery for a recurrent hiatal hernia with dysphagia: case report. Chirurgia. 2014;109(4):538-41. PubMed PMID: 25149620.

2. Cano-Valderrama O, Marinero A, Sanchez-Pernaute A, Dominguez-Serrano I, Perez-Aguirre E, Torres AJ. Aortic injury during laparoscopic esophageal hiatoplasty. Surgical endoscopy. 2013;27(8):3000-2. doi: 10.1007/s00464-013-2826-6. PubMed PMID: 23436085.

3. De Moor V, Zalcman M, Delhaye M, El Nakadi I. Complications of mesh repair in hiatal surgery: about 3 cases and review of the literature. Surgical laparoscopy, endoscopy & percutaneous techniques. 2012;22(4):e222-5. doi: 10.1097/SLE.0b013e318253e440. PubMed PMID: 22874707.

4. Makarewicz W, Jaworski L, Bobowicz M, Roszak K, Jaroszewicz K, Rogowski J, et al. Paraesophageal hernia repair followed by cardiac tamponade caused by ProTacks. The Annals of thoracic surgery. 2012;94(4):e87-9. doi: 10.1016/j.athoracsur.2012.03.107. PubMed PMID: 23006720.

5. Rodriguez-Lago I, Munoz-Navas M, Carrascosa J, Betes M, Valenti V, Rotellar F. Successful endoscopic mesh removal after laparoscopic Nissen fundoplication. Revista espanola de enfermedades digestivas : organo oficial de la Sociedad Espanola de Patologia Digestiva. 2012;104(11):605-6. PubMed PMID: 23368653.

6. Paz YE, Vazquez J, Bessler M. Cardiac tamponade as a complication of laparoscopic hiatal hernia repair: case report and literature review. Catheterization and cardiovascular interventions : official journal of the Society for Cardiac Angiography & Interventions. 2011;78(5):819-21. doi: 10.1002/ccd.23178. PubMed PMID: 21990104.

7. Carpelan-Holmstrom M, Kruuna O, Salo J, Kylanpaa L, Scheinin T. Late mesh migration through the stomach wall after laparoscopic refundoplication using a dual-sided PTFE/ePTFE mesh. Hernia : the journal of hernias and abdominal wall surgery. 2011;15(2):217-20. doi: 10.1007/s10029-010-0633-8. PubMed PMID: 20130942.

8. Frantzides CT, Welle SN. Cardiac tamponade as a life-threatening complication in hernia repair. Surgery. 2012;152(1):133-5. doi: 10.1016/j.surg.2011.08.009. PubMed PMID: 21944871.

9. Hazebroek EJ, Leibman S, Smith GS. Erosion of a composite PTFE/ePTFE mesh after hiatal hernia repair. Surgical laparoscopy, endoscopy & percutaneous techniques. 2009;19(2):175-7. doi: 10.1097/SLE.0b013e3181a11926. PubMed PMID: 19390288.

10. Kepenekci I, Turkcapar AG. Mesh erosion as a complication of laparoscopic fundoplication with prosthetic hiatal closure: report of a case. Surgical laparoscopy, endoscopy & percutaneous techniques. 2009;19(2):e51-4. doi: 10.1097/SLE.0b013e3181979a45. PubMed PMID: 19390264.

11. Stadlhuber RJ, Sherif AE, Mittal SK, Fitzgibbons RJ, Jr., Michael Brunt L, Hunter JG, et al. Mesh complications after prosthetic reinforcement of hiatal closure: a 28-case series. Surgical endoscopy. 2009;23(6):1219-26. doi: 10.1007/s00464-008-0205-5. PubMed PMID: 19067074.

12. Tatum RP, Shalhub S, Oelschlager BK, Pellegrini CA. Complications of PTFE mesh at the diaphragmatic hiatus. Journal of gastrointestinal surgery : official journal of the Society for Surgery of the Alimentary Tract. 2008;12(5):953-7. doi: 10.1007/s11605-007-0316-7. PubMed PMID: 17882502.

13. Bonavina L, Bona D, Saino G, Clemente C. Pseudoachalasia occurring after laparoscopic Nissen fundoplication and crural mesh repair. Langenbeck's archives of surgery / Deutsche Gesellschaft fur Chirurgie. 2007;392(5):653-6. doi: 10.1007/s00423-007-0191-4. PubMed PMID: 17530282.

14. Hergueta-Delgado P, Marin-Moreno M, Morales-Conde S, Reina-Serrano S, Jurado-Castillo C, Pellicer-Bautista F, et al. Transmural migration of a prosthetic mesh after surgery of a paraesophageal hiatal hernia. Gastrointestinal endoscopy. 2006;64(1):120; discussion 1. doi: 10.1016/j.gie.2006.01.034. PubMed PMID: 16813816.

15. Muller-Stich BP, Linke G, Leemann B, Lange J, Zerz A. Cardiac tamponade as a life-threatening complication in antireflux surgery. American journal of surgery. 2006;191(1):139-41. doi: 10.1016/j.amjsurg.2005.08.006. PubMed PMID: 16399125.

16. Thijssens K, Hoff C, Meyerink J. Tackers on the diaphragm. Lancet. 2002;360(9345):1586. doi: 10.1016/S0140-6736(02)11530-3. PubMed PMID: 12443612.

17. Baladas HG, Smith GS, Richardson MA, Dempsey MB, Falk GL. Esophagogastric fistula secondary to teflon pledget: a rare complication following laparoscopic fundoplication. Diseases of the esophagus : official journal of the International Society for Diseases of the Esophagus / ISDE. 2000;13(1):72-4. PubMed PMID: 11005336.

18. Kemppainen E, Kiviluoto T. Fatal cardiac tamponade after emergency tension-free repair of a large paraesophageal hernia. Surgical endoscopy. 2000;14(6):593. doi: 10.1007/s004640000138. PubMed PMID: 11265062.

19. Farlo J, Thawgathurai D, Mikhail M, Yaker K, Sullivan J, Morgan E. Cardiac tamponade during laparoscopic Nissen fundoplication. European journal of anaesthesiology. 1998;15(2):246-7. PubMed PMID: 9587736.
